# Antioxidant metabolism insights into ripening and senescence delay of green pepper fruit through the salicylic acid preharvest treatment

**DOI:** 10.3389/fpls.2025.1475068

**Published:** 2025-03-19

**Authors:** Alicia Dobón-Suárez, María Gutiérrez-Pozo, Vicente Serna-Escolano, María J. Giménez, Daniel Valero, María Serrano, María E. García-Pastor, Pedro J. Zapata

**Affiliations:** ^1^ Department of Agri-Food Technology, Institute for Agri-Food and Agro-Environmental Research and Innovation (CIAGRO), University Miguel Hernández, Alicante, Spain; ^2^ Department of Applied Biology, Institute for Agri-Food and Agro-Environmental Research and Innovation (CIAGRO), University Miguel Hernández, Alicante, Spain

**Keywords:** *Capsicum annuum* L., quality losses, bioactive compounds, antioxidant capacity, antioxidant enzymes, relative gene expression

## Abstract

**Introduction:**

The systematic investigation of the biochemical and molecular bases of salicylic acid (SA) in the postharvest physiological process of green pepper fruit remains unclear.

**Methods:**

Accordingly, this study aims to analyze the effects of 0.5 mM-SA preharvest treatments, applied by foliar spraying or irrigation, on the ripening and senescence of green pepper fruit for 28 days of storage at 7 °C.

**Results:**

The study revealed that the preharvest application of SA, either by foliar spraying or irrigation, significantly delayed losses of weight, firmness and color during postharvest. Additionally, both treatments increased the total soluble solids and total acidity content, which lead to a significantly reduced ripening index after storage. These results were evidenced by a slowing down of the ripening and senescence processes, accompanied by the stimulation of the antioxidant enzymes in those SA-treated green pepper fruits. Furthermore, a significant increase in chlorophylls, phenolics, ascorbic acid and dehydroascorbic acid content was observed. The SA treatments also enhanced the total antioxidant activity, in both hydrophilic and lipophilic phases. These positive effects were mediated by the upregulation of the relative response of the *CaAPX, CaPOD, CaPAL, CaDHAR2* genes at harvest.

**Discussion:**

These findings reinforce the existing knowledge gap regarding the impact of foliar spraying or irrigation SA on the intricate interplay between metabolites and genes related to the antioxidant system in regulating the bell pepper fruit ripening and senescence. The impact of both applications exhibited comparable results; however, the irrigation was identified as the most advantageous due to its ease applicability and cost effectiveness in comparison.

## Graphical Abstract

An antioxidant metabolism approach delaying ripening and senescence of green pepper fruit by applying foliar or irrigation salicylic acid (SA) preharvest treatment.

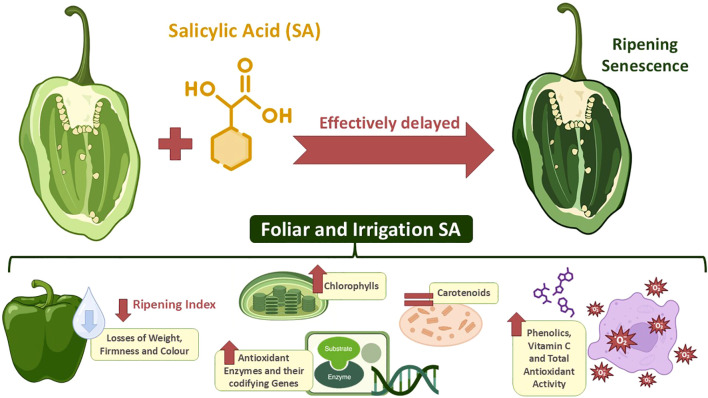

## 1 Introduction

Bell pepper (*Capsicum annuum* L.) is an economically important vegetable crop which has a recent worldwide popularity among consumers in human diet due to its nutritional value, as an excellent source of biologically active compounds (vitamins, carotenoids, flavonoids, phenolic acids and other phytochemicals) with health-related properties, the crispness, and the versatility to be consumed as a fresh vegetable in salads, cooked meals or dehydrated for spices (Marín et al., 2004; Navarro et al., 2006; Serrano et al., 2010; Raybaudi-Massilia et al., 2017; Soare et al., 2017; Ge et al., 2020). Consumers have become more critical in the last decade with their purchasing decisions which are commonly focused not only in physical and sensory traits, such as color, size, pericarp thickness, firmness and flavor, but also in nutritional and nutraceutical characteristics (Rodríguez-Burruezo et al., 2010; Jiménez-García et al., 2018). In Spain, the total production of chili peppers and peppers (*Capsicum annuum* L. and *Piper nigrum* L, respectively) has steadily increased to more than 1.58-fold over the past 10 years, reaching over 1.5 million tons in the 2022 season (FAOSTAT, 2024). The primary postharvest challenges that result in substantial quality deterioration and diminished acceptability of bell peppers are as follows: Water loss, which leads to significant softening and shrinkage due to turgor pressure loss, starch degradation, and chemical modifications in the cell wall related to pectin by the action of softening enzymes, such as polygalacturonase (PG), pectin methyl esterase (PME), cellulase, and ß-galactosidase (Li Z. et al., 2024). On the other hand, peppers can experience chilling injury (CI) when stored at temperatures below 7-10°C. This leads to symptoms such as surface pitting, watery stains, browning of the seed and calyx, and fruit decay. This, in turn, can result in reduced marketability (El-Ramady et al., 2015; Charoenphun et al., 2024). Finally, there are pathological disorders, for example grey mold, which is mainly caused by *Botrytis cinerea* (Cheema et al., 2018).

Nowadays, global demand for high-quality vegetable products is rapidly increasing. However, climate change is negatively affecting agricultural areas and water resources which are decreasing (Sobczak et al., 2023). Bell pepper crop is sensitive to temperature fluctuations during the developmental and growth cycle, showing a lack of tolerance to high temperature since its fruit setting is drastically reduced when the day temperature rises above 32°C and/or the night temperature is above 20°C (Erickson and Markhart, 2002). This abiotic stress in the plant induces the production and accumulation of reactive oxygen species (ROS) leading to membrane breakdown and cellular turgor loss that can prevent plant growth and development (Preet et al., 2023). The process of scavenging ROS in plants as part of the antioxidant defense system and osmoprotectants comprises various antioxidant enzymes, including superoxide dismutase (SOD), which converts free superoxide (O_2_) radicals to hydrogen peroxide (H_2_O_2_) and oxygen, ascorbate peroxidase (APX), catalase (CAT), and peroxidase (POD). The latter enzymes have shown to play a pivotal role in the detoxification of H_2_O_2_ through its decomposition into water and oxygen. Furthermore, the role of radical scavenging metabolites in ROS scavenging mechanisms is also of significance. In this sense, different phytohormones can positively influence the crop yield and reduce negative environmental impacts. On the other hand, fruit senescence and postharvest disease infection can result in quality and economic losses (Zhang and Jiang, 2019). Some plant hormones have been widely studied to improve the postharvest quality of fruit and vegetables (Zhang et al., 2020).

As the major endogenous component in signal transduction systems, SA plays an efficient role in plant growth and development, flowering, and fruit ripening, as well as in regulating photosynthesis (Natasha et al., 2020; Hadjipieri et al., 2021). On the other hand, SA is crucial in stimulating the systemic acquired resistance (SAR) in plants by regulating numerous biochemical and physiological functions related to tolerance to both biotic and abiotic stresses and modifying the antioxidant system (Khan et al., 2015). Therefore, SA and its derivatives elicit a wide range of metabolic and physiological processes in plants, which have great potential in reducing postharvest losses in horticultural crops. SA leads to the synthesis of proteins affecting several metabolic processes by regulating their gene expression (Janda et al., 2020). The mechanisms by which SA generates these improvements could be related to the protection of cell membranes, the increase in carbon metabolism, and antioxidant system, and the regulation of stress defense proteins (Kang et al., 2012; Sharma et al., 2017). However, its specific action mechanism is still not well understood. Indeed, different studies have demonstrated the hormonal interactions between SA and jasmonic acid (JA) and several different stress-link compounds taking place under abiotic stresses, highlighting the complexity of hormonal signaling cascades (Dempsey and Klessig, 2017; Devireddy et al., 2021; Huntenburg et al., 2022). Furthermore, the attenuating effects of SA in plants depend on the concentration used, the method of application, and the plant development stage (Nóbrega et al., 2020).

In recent years, the foliar application of exogenous SA to crops has shown to be effective in the regulation of biotic and abiotic stresses, increasing the yield of green pepper fruit by reducing stress-induced growth inhibition as well as fruit quality traits (Elwan and El-Hamahmy, 2009; Jiménez-García et al., 2018; Ibrahim et al., 2019; Veloso et al., 2021; Dobón-Suárez et al., 2021b; Ghahremani et al., 2023; Sobczak et al., 2023; Preet et al., 2023; Rodrigues da Silva et al., 2023). In addition, SA treatment was found to alleviate chilling injury in pepper fruits through enhancing antioxidant metabolism, fatty-acid desaturation efficiency and water retention (Fung et al., 2004; Ge et al., 2020; Hanaei et al., 2022). However, appropriate concentrations and methods of elicitor application need to be determined to improve the effectiveness of this practice under different growing conditions (Munshi et al., 2020; Sobczak et al., 2023). A recent review concluded that preharvest spraying provides better results than postharvest treatments, but the specific results need deeper research to be conducted focusing on the effect of spraying frequency time and growing environment on postharvest storage quality of fruit (Chen S. et al., 2023). Furthermore, Chen S. et al. (2023) stated that there is a lack of information about the metabolic mechanism of exogenous treatments with SA and its derivatives affecting fruit quality parameters, which is an emerging field that needs to be explored. In fact, most of the metabolomic and transcriptomic studies of SA available are related to the functions, biosynthesis or transcriptional regulations of this plant hormone for establishing resistance to many pathogens in plants (Ding and Ding, 2020). For instance, exogenous SA application bolstered resistance to *Colletotrichum viniferum* (Lin et al., 2024), *Ralstonia solanacearum* (Li N. et al., 2024), *Podosphaera pannosa* (Yang et al., 2022), *Xanthomonas campestris pv. campestris* (Sun et al., 2022), *Colletotrichum* (Shi et al., 2019), cucumber green mottle mosaic virus (Liu et al., 2023), and *Penicillium expansum* (Zhang et al., 2024) in grapes, tomato, roses, cabbage, tea, watermelon, and apples, respectively. Recently, Perez-Aranda et al. (2024) demonstrated that the expression of the *CaPR1* (Pathogenesis-related protein 1) in *Capsicum annuum* seedlings, a marker gene employed as indicator of SA pathways activation, is down-regulated with SA elicitation (1, 2.5 and 5 mM) and the cross-talk between jasmonic acid/ethylene and SA mediated signal pathways for the regulation of this gene. Other studies have revealed that the transcriptional activation of SA signaling pathway, rather than its biosynthesis, plays a crucial role in the chilling and freezing tolerance of cucumber and potato fruit, respectively (Sim et al., 2024; Chen L. et al., 2023). However, Nguyen et al. (2024) reported that the ripening quality of mango mirrored the induced SA and jasmonic acid (JA) endogenous levels after liquid methyl salicylate (MeSA) fumigants in postharvest and correlated with the high expression of biosynthetic-related genes. In this sense, in-depth study of both foliar spraying and irrigation methods to pepper plants has received little scientific attention, and the systematic investigation of the mechanism and molecular bases of SA effects in the postharvest physiological process of green pepper fruit remains unclear, which restricts the possibilities for improvement of green pepper fruit quality using preharvest elicitation tools. Therefore, this paper aims to compare the effect of SA preharvest treatment applied by foliar spraying or irrigation on ripening and senescence of green pepper fruit during postharvest storage from an antioxidant metabolism approach to provide deep insight into a practical application of SA on extending the storage shelf-life.

## 2 Materials and methods

### 2.1 Plant materials, treatments and experimental design

Pepper plants (*Capsicum annuum* L., ‘Lamuyo’ type), ‘Herminio’ cultivar, were planted in January 2021 in a commercial plot growing under plastic-roofed greenhouse located in El Raal (Murcia, Spain). The optimal concentration of salicylic acid (SA) was chosen according to the best results observed in our latest study about the evaluation of SA foliar application on crop yield and quality parameters of green pepper fruit during 21 days of storage at 7°C (Dobón-Suárez et al., 2021a). In this study, SA applied at 0.5 mM showed the best results since this treatment increased crop yield, in terms of kg per plant, number of fruits harvested per plant, average fruit weight, fruit quality parameters and bioactive compound content at harvest. In addition, this treatment delayed losses of physio-chemical and functional traits that normally occur during postharvest storage of pepper fruit at non-chilling temperatures, maintaining fruit quality after 21 days of storage. Finally, SA preharvest treatment applied at 0.5 mM was the most effective tool to induce pepper fruit tolerance against decay incidence during storage. Therefore, SA was applied in the present study at 0.5 mM following two different commercial practices: *1)* Foliar spraying [Foliar SA] and *2)* Irrigation [Irrigation SA], while the control plants were treated only with distilled water as a spray [Control]. SA reagent (CAS Number: 69-72-7) was purchased from Sigma (Sigma-Aldrich, Madrid, Spain). Solutions for all treatments were supplemented with Tween 20 [0.05% (*v/v*)]. The foliar spray was carried out with a manual pump, while the root application was performed in the automatic irrigation system was carried out automatically. Plants received irrigation and fertilization according to normal agricultural practices designed by the company for the short-term crop cycle of ‘Lamuyo’ pepper type, in which rockwood was used as the soil substrate and drip irrigation and optimal nutrient levels were applied. The soil texture was sandy loam with a pH of 7.50.

The experiment was conducted from February to July 2021. The experimental design was completely randomized. Thus, 135 pepper plants were selected and distributed in randomized complete block design with nine replicates or blocks in total. Each treatment was performed in three blocks (n = 3) of 15 plants (45 plants per treatment). Seven exogenous SA applications by foliar spraying or irrigation throughout the crop cycle were performed in the morning (8-9 a.m.), the first treatment being applied before the beginning of the flowering stage. Treatments were applied seven times at a 21-d interval until the harvest date with a total amount of SA supplied of 0.48 g L^-1^. The equidistance among application dates was *ca.* 21 days due to a staggered flowering cycle, except for the last application that was performed close to the last commercial harvest, being chosen based on the crop cycle duration of this pepper cultivar and our previous experience (Dobón-Suárez et al., 2021a). Application dates of treatments (Control, Foliar SA, and Irrigation SA) throughout the developmental and growth cycle of ‘Herminio’ green pepper fruit were as follows: T1 (22 February), T2 (15 March), T3 (29 March), T4 (19 April), T5 (17 May), T6 (7 June) and T7 (10 July). Pepper fruits were harvested at the commercial harvest stage when green pepper had reached the phenological stage suitable for its consumption (Dobón-Suárez et al., 2021b). A total of 10 harvest dates throughout the growth cycle were performed according to a staggered production and the commercial criteria of harvesting green pepper fruit established by the company. The harvest dates started from April until July: 6 April, 20 April, 4 May, 14 May, 26 May, 4 June, 16 June, 26 June, 6 July and 17 July. The mean temperature for each month was recorded: April (16.00°C), May (19.30°C), June (19.70°C) and July (27.20°C), using a station close to the experimental greenhouse (38°2’2.64” North, 1°1’18.9” West). Relative humidity (RH) fluctuated between 66 to 89% during the experiment. The crop yield was measured in terms of accumulative crop yield, expressed as kg per plant and number of peppers harvested per plant, for each harvest date along the crop cycle and blocks designed per treatment. In addition, the average fruit weight (g) was calculated by weighing and counting all harvested pepper fruits individually, according to Dobón-Suárez et al. (2021a). These results are presented in **Supplementary Figure 1**. The uniform-sized pepper fruits harvested on 20 April were immediately transferred to the research laboratory of Postharvest Group of Fruit and Vegetables and then, they were graded for their uniformity in shape and color, and those fruits free from visual defects and blemishes were selected to carry out a postharvest storage experiment.

For each treatment 90 peppers fruits similar in shape, size and color were selected and weighted individually and stored at 7°C and 85% of RH. Thus, 18 pepper fruits were analyzed at harvest (day 0) and 72 pepper fruits in total were stored for each treatment during 21 days of storage. For the postharvest storage experiment, pepper fruits were analyzed after 7, 14, 21 and 28 days of storage. Specifically, 90 pepper fruits were used in total for the analyses of each treatment in the four sampling dates (0, 7, 14, 21 and 28 storage days). For each sampling date, weight loss, firmness, color (hue°), total soluble solids (TSS), total acidity (TA) and ripening index (RI) were measured as quality parameters of green pepper fruits during postharvest storage. From a metabolomic approach, the content of chlorophyll a and b, total phenolics, total carotenoids, ascorbic acid (AA) and dehydroascorbic acid (DHA) was quantified at harvest and after 28 days of storage at 7°C in freeze-dried samples composed of both flesh and skin tissues. Furthermore, the hydrophilic-total antioxidant activity (H-TAA), lipophilic-total antioxidant activity (L-TAA) and the antioxidant enzymes activities of ascorbate peroxidase (APX), catalase (CAT) and peroxidase (POD) were also determined in freeze-dried material at harvest and after 28 days of postharvest storage. Finally, a genetic approach was also addressed since the relative expression of *CaAPX* [*L*-ascorbate peroxidase (APX) gene], *CaCAT* [catalase (CAT) gene], *CaPOD* [peroxidase (POD) gene], *CaPAL* [phenylalanine ammonia-lyase (PAL) gene] and *CaDHAR2* [dehydroascorbate reductase 2 gene] genes was also analyzed in freeze-dried samples of green pepper fruits at harvest and at the end of the storage period. All analyses were performed on 3 replicates of 6 fruits for each treatment and sampling date studied (18 pepper fruits in total).

### 2.2 Evaluation of quality parameters during postharvest storage

#### 2.2.1 Weight loss, firmness and color (hue°) of green pepper fruits

Green pepper fruits were initially weighed at harvest (day 0) and after 7, 14, 21 and 28 days of storage. The difference between the initial and final weight of pepper fruit was considered as accumulative weight loss during each storage interval and was expressed as a percentage (%) on a fresh weight basis with respect to pepper fruit weight at harvest. Firmness was evaluated individually in each pepper fruit as deformation force using a digital TX-XT2i Texturometer (Stable Microsystems, Godalming, UK). The machine had a flat steel plate to measure the equatorial fruit diameter and to apply a force that achieved a 5% deformation of its diameter, according to the protocol described by Dobón-Suárez et al. (2021a). Results were expressed as a force-deformation ratio (N mm^-1^). After that, 6 pepper fruits from each replicate (18 peppers from each treatment) were used to measure individually the color. Surface color changes of green pepper fruits were reported in hue angle (hue°) parameter (arctan b*/a*), according to García-Pastor et al. (2021). It was measured at three points of the fruit equatorial diameter by using a Minolta Colorimeter CFRC400 (Minolta Camera Co., Kantō, Tokio, Japan).

#### 2.2.2 Total soluble solids, total acidity and ripening index of green pepper fruits

A homogeneous sample was prepared from each replicate and treatment by blending the fruit in a blender. The sample was thoroughly mixed, and a few drops were taken on prism of a portable digital refractometer (Atago PR-101, Atago Co., Ltd., Tokyo, Japan) to measure the content of total soluble solids (TSS) of each sample in duplicate at 20°C. Results were expressed as g kg^-1^ in fresh weight basis (FW). As described by Dobón-Suárez et al. (2021a), total acidity (TA) was determined in duplicate from the same sample by titrating 1 mL of diluted juice in 25 mL of distilled H_2_O with 0.1 N NaOH up to a pH of 8.10 using an automatic titration (785 DMP Titrino, Metrohm, Burladingen, Germany). Results were expressed as g of malic acid equivalent kg^-1^ FW. Ripening index (RI) was then calculated as the ratio of TSS/TA.

### 2.3 Metabolomic analysis at harvest and after 28 days of storage

#### 2.3.1 Quantification of bioactive compounds

Bioactive compounds were quantified in green pepper fruits at harvest (day 0) and after 28 days of storage at 7°C. The chlorophyll a and b, and total carotenoids were extracted according to previously described methods (Knee, 1972; Xie et al., 2023) with some modifications. Approximately 0.20 g of fine freeze-dried powder for the three biological replicates (n = 3) were manually grounded in a mortar and pestle and mixed with 5 mL of acetone extract solution containing 0.1% BHT to prevent the pigment from oxidizing. Next, the mixed extraction was ultrasonically extracted for 15 min and then centrifuged at 10,000 *g* for 10 min at 4°C to obtain the supernatant. The sample was repeatedly extracted until the residue was colorless. Acetone solution containing 0.1% BHT was used for a constant volume of collected supernatants (25 mL) to the subsequent estimation of chlorophyll and total carotenoid contents. Based on the methods reported by Lichtenthaler and Wellburn (1983), the absorbance of the extracts was detected at 470, 645, and 662 nm by spectrophotometric absorbance (UV-1900i-UV-VIS Spectrophotometer, Shimadzu Corporation, Germany) to quantify the chlorophyll and total carotenoid contents, which were calculated from the equations: C_a_ = 11.75A_662_ - 2.35A_645_, C_b_ = 18.6lA_645_ - 3.96A_662_ and C_TC_ = (1000A_470_ - 2.27C_a_ − 81.4C_b_)/227. C_a_, C_b_ and C_TC_ indicate the content of chlorophyll a, chlorophyll b and total carotenoids (g kg^-1^ DW), respectively. The total chlorophyll content was calculated as the sum of the chlorophyll a and chlorophyll b contents (g kg^-1^ DW). A_662_, A_645_, A_470_ represent absorbances at 662 nm, 645 nm and 470 nm, respectively.

Ascorbic (AA) and dehydroascorbic (DHA) acids were measured in the freeze-dried powder for each replicate (n = 3), according to the methodology of Peña-Estévez et al. (2016) with slight modifications. Thus, 0.20 g of fine powder was homogenized manually in a mortar and pestle and mixed with 5 mL of a methanol: water (5:95) solution containing 0.1 mM citric acid, 0.05 mM ethylenediamine tetracetic acid disodium salt, and 4 mM NaF. Then, the extract was filtered through a four-layer cheesecloth and the pH was adjusted to 2.35-2.40 with 2 N HCl. The mixed extraction was centrifuged at 10,000 *g* for 15 min at 4°C and the supernatant was purified through a methanol-activated C18 cartridge (Sep-Pak cartridges C18, Waters, Dublin, Ireland) and filtered through a 0.45 μm PFTE filter. For DHA derivatization, 750 μL of extract was mixed with 250 μL of 7.7 M 1,2-phenylenediamine in an HPLC amber vial. The mixture was allowed to react for 37 min and then 20 μL were injected onto a Luna (250 mm × 4.6 mm, 5 μm particle size) C18 column (Phenomenex, Macclesfield, UK) with a C18 security guard (4.0 mm × 3.0 mm) cartridge system (Phenomenex) using an HPLC system (1200 Infinity series, Agilent Technologies, Waldbronn, Germany). The mobile phase was 50 mM KH_2_PO_4_ containing 5 mM hexadecyl trimethylammonium bromide and 5% methanol (pH 4.59) with an isocratic flow of 1 mL min^-1^. Absorbance was recorded at 261 nm for AA (Rt = 9.4 min) and at 348 nm for DHA (Rt = 4.5 min), and both values were quantified by comparison with AA and DHA standard areas (Sigma-Aldrich, Darmstadt, Germany). Total vitamin C was defined as the sum of both AA and DHA content. Results (mean ± SE) were expressed as g kg^-1^ of dry weight (DW).

The quantification of total phenolic compounds (TPC) was carried out from the hydrophilic phase obtained in the total antioxidant activity extraction, as previously described by Dobón-Suárez et al. (2021b). Briefly, 5 g of green pepper fruits were homogenized with 10 mL of 50 mM phosphate buffer pH = 7.8 and 5 mL of ethyl acetate using a homogenizer (Ultraturrax, T18 basic, IKA, Berlin, Germany) for 30 s. The extracts were centrifuged at 10,000 *g* for 10 min at 4°C and the supernatant was used to quantify the total phenolic content in duplicate in each extract by using the Folin-Ciocalteu reagent (Sayyari et al., 2011). Results were expressed as g gallic acid equivalent (GAE) kg^-1^ of fresh weight (FW) and are the mean ± SE of three replicates.

#### 2.3.2 Total antioxidant capacity: hydrophilic and lipophilic fractions

Total antioxidant capacity was determined in both hydrophilic and lipophilic fractions at harvest (day 0) and after 28 days of storage at 7°C after cutting the pepper fruit, removing its peduncle and seeds, and being frozen with liquid N_2_ and stored at -20°C. As previously reported in green pepper fruit (Dobón-Suárez et al., 2021b), 5 g of frozen samples were extracted with 10 mL of 50 mM phosphate buffer pH = 7.8 and 5 mL of ethyl acetate. The extracts were homogenized using a homogenizer (Ultraturrax, T18 basic, IKA, Berlin, Germany) for 30 s and then, centrifugated at 10,000 *g* for 15 min at 4°C. Both upper and lower fractions were used to quantify the hydrophilic (H-TAA) and lipophilic (L-TAA) total antioxidant activity, respectively. Both antioxidant fractions were measured in duplicate using a reaction mixture in which ABTS^+^ radicals are generated and monitored at 730 nm. Results were expressed as g of Trolox equivalent (TE) kg^-1^ FW and are the mean ± SE (n = 3).

#### 2.3.3 Assays of antioxidant enzymes

The antioxidant activity of ascorbate peroxidase (APX), catalase (CAT) and peroxidase (POD) enzymes were also determined in freeze-dried powder (flesh + skin tissues) maintained at -80°C both at harvest (day 0) and after 28 days of postharvest storage. APX, CAT and POD enzymes were extracted by homogenizing 0.20 g of fine powder with 5 mL of phosphate buffer 50 mM, pH 6.8, containing 1% (*w/v*) of polyvinylpyrrolidone (PVP) and ethylenediamine-tetracetic acid 1 mM. After centrifugation at 10,000 *g* for 15 min at 4°C, the supernatant was used to quantify each enzyme activity in duplicate, as reported elsewhere (García-Pastor et al., 2021). Antioxidant enzyme activities were expressed as units of enzymatic activity (U min^-1^ g^-1^) of dry weight (DW) with one enzymatic unit (U) being defined as a 0.01 decrease of ascorbate at 290 and 240 nm min^-1^ for APX and CAT, respectively, and a 0.01 increase of absorbance at 470 nm min^-1^ for POD. Results were the mean ± SE of three replicates (n = 3).

### 2.4 Gene expression analysis at harvest and after 28 days of storage

Plant RNA was extracted from 0.03 g of freeze-dried samples (flesh + skin tissues of green pepper fruit) to analyze the relative expression of targeted genes at harvest (day 0) and after 28 days of storage. Total RNA was extracted using the RNeasy Plant Mini Kit (Qiagen, Dusseldorf, Germany) according to manufacturer’s instructions, in which a DNase treatment was recommended on the eluted RNA by using Baseline-ZERO DNase (Epicentre/Lucigen USA). RNA quantification was carried out by the spectrophotometric absorbance using an Implen Nanophotometer^®^ (IMPLEN, Munich, Germany). RNA extracts were maintained at -80°C. The single-strand cDNA was synthesized from 500 ng of total RNA using the PrimeScript RT Master Mix (Perfect Real Time) kit (Takara Bio, Japan) in a Mastercycler Nexus X2 (Eppendorf, Germany) PCR machine, following the manufacturer’s protocol. This synthesis and the subsequent qPCR for the expression of the targeted genes were carried out by Genomic Centre of the Complutense University of Madrid (Madrid, Spain). Total RNA (15-40 ng per reaction) from three biological replicates and treatments was used as the template for the OneStep qPCR reactions.

Two housekeeping genes, ubiquitin (*CaUBI*) and actin (*CaACT*), were selected as reference genes in *Capsicum annuum* L (Li et al., 2020; Seo et al., 2020; Ge et al., 2020). The relative expression of five genes was evaluated as targeted genes: *L*-ascorbate peroxidase gene (*CaAPX*), catalase gene (*CaCAT*), peroxidase gene (*CaPOD*), phenylalanine ammonia-lyase gene (*CaPAL*) and dehydroascorbate reductase 2 gene (*CaDHAR2*). The gene-specific primers used are listed in **Table 1**. Gene sequences were obtained from the National Center for Biotechnology Information (https://www.ncbi.nlm.nih.gov/). The amplicon length for each primer was 123 pb for *CaUBI*, 130 pb for *CaACT*, 238 pb for *CaAPX*, 104 pb for *CaCAT*, 131 pb for *CaPOD*, 676 pb for *CaPAL* and 136 pb for *CaDHAR2*. The primers for qRT-PCR (**Table 1**), purchased from Merck (Sigma-Aldrich, Darmstadt, Germany). The reactions were prepared in duplicate on 384-well plates using PowerUp SYBR^®^ Green Master Mix (Applied Biosystems, California) with the primers at a concentration of 300 nM in a reaction volume of 10 µL. The qPCR analysis was performed in a QuantStudio™ 7 Flex Real-Time PCR System (Applied Biosystems, California) with an initial step at 95°C for 10 min followed by 40 cycles of 95°C for 15 s and 60°C for 1 min. Additionally, the quality of amplicons was controlled by a melt curve analysis step showing no side products. Data obtained from the qPCR was treated with the QuantStudio Reat-Time PCR software (Applied Biosystems, California). Relative targeted gene expression in treated green pepper fruit was normalized using the expression levels of the *CaUBI* and *CaACT* genes and was calculated regarding control fruit using three biological replicates (n = 3).

**Table 1:** Genetic details of primers for the reference and targeted genes^ϯ^.

Gene	Forward Primer Sequence (5’-3’)	Reverse Primer Sequence (5’-3’)	NCBI Reference
***CaUBI***	GGCATGTCTGGGACTTTTGC	AGACCCGTTCCTTGACAACC	AY486137.1
***CaACT***	ACCCTGTGCTTCTCACTGAAG	GCATAAAGAGACAACACCGCC	AY572427.1
***CaAPX***	ACTGGTGGACCGAATGGTTC	GTAACCGCCCTTCCTTTGGA	NM_001324587.1
***CaCAT***	TATCCGATCCCCGAGCAACT	CACAGTGAGACGAGAAGCG	AF227952.1
***CaPOD***	AACAGGGAAACCCGAATGGG	TTTGGTGCAGCCCTCTTCTC	FJ596178
***CaPAL***	ATGCTCTTAGAACGTCGCCC	AAGACGTATTCCCTGTCCACG	NM_001325423
***CaDHAR2***	GTTGATTTGAGCTTGGCCCC	TCTGGAAAGACTCACGCTCG	KJ950368.1

**^ϯ^** Based on National Center for Biotechnology Information (https://www.ncbi.nlm.nih.gov/).

### 2.5 Statistical analysis

The experiment was conducted using a randomized design with three replicates (n = 3). Data are expressed as mean ± standard error (SE). Statistical comparisons of the means were performed using one-way analysis of variance (ANOVA) of SPSS software package v. 17.0 for Windows (SPSS, 2001, IBM Corporation, Armonk, NY, USA). The source of variation was treatments. Mean separation was analyzed using Tukey’s HSD test to determine whether the differences among treatments were significant at *p* < 0.05. The heatmap analysis was conducted with Microsoft Excel^®^ for Windows (Excel, 2016, Microsoft Corporation, Redmond, Washington, USA).

## 3 Results

### 3.1 Foliar and irrigation SA delays quality losses during postharvest storage

**Figure 1:**
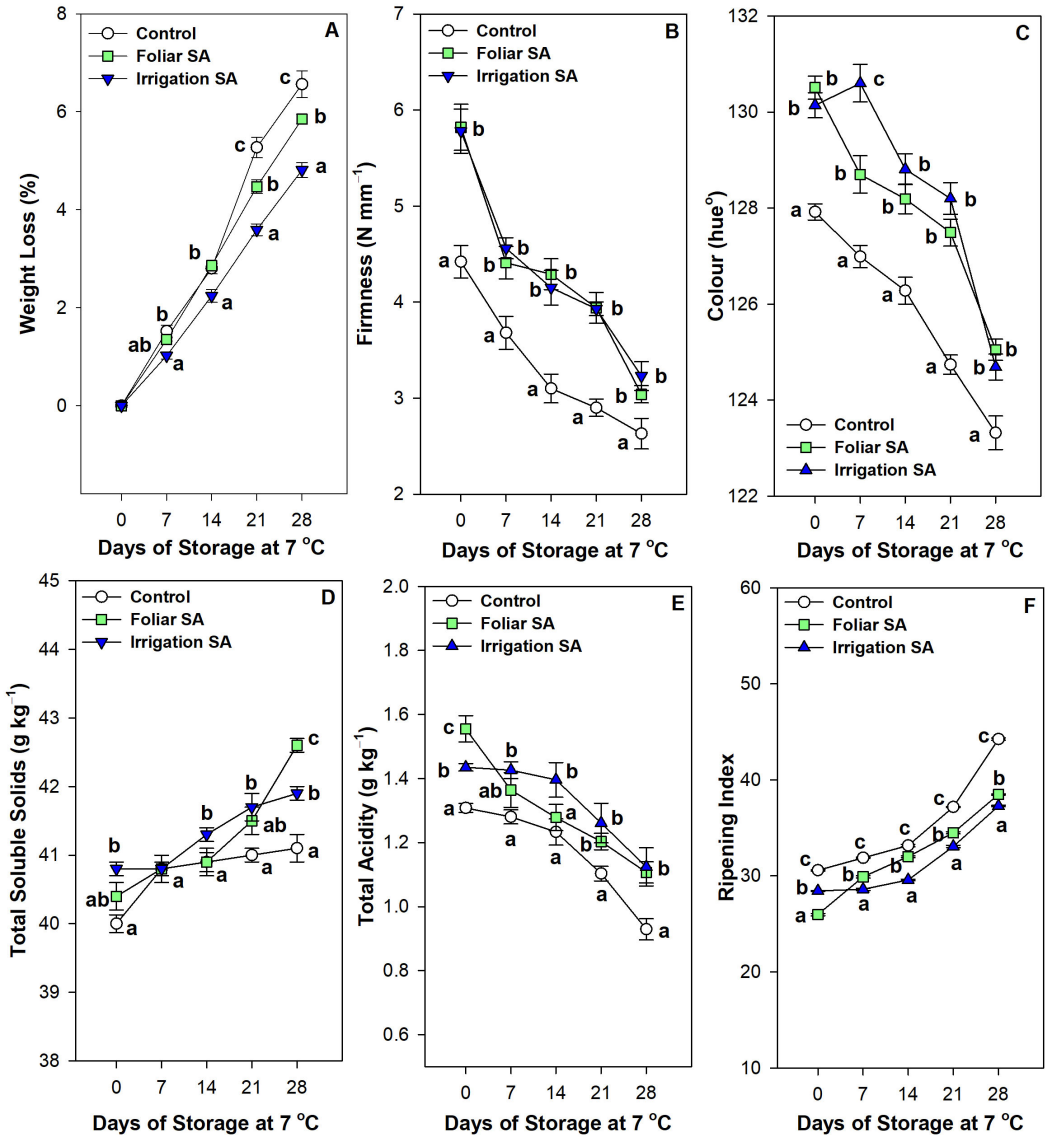
Effect of salicylic acid (SA) applied by foliar spraying [Foliar SA] and irrigation [Irrigation SA] on weight loss (%) **(A)**, firmness (N mm^-1^) **(B)**, color (hue°) **(C)**, total soluble solids (g kg^-1^) **(D)**, total acidity (g kg^-1^) **(E)** and ripening index **(F)** of green pepper fruit during 28 days of storage at 7°C. Different lowercase letters indicate significant differences at p < 0.05 according to Tukey’s HSD test among treatments for each sampling date and parameter tested.

SA applied by foliar spraying and irrigation significantly reduced (*p* < 0.05) weight loss in green pepper fruit, ‘Herminio’ cv., after 28 days of storage at 7°C compared to untreated fruits. Specifically, irrigation SA showed a 4.81% of weight loss than the 6.56% achieved in control treatment, with a 1.36-fold reduction, at 28 storage days (**Figure 1A**). SA treated green pepper fruits showed the highest firmness levels at harvest (≈ 5.80 N mm^-1^) compared with control (4.42 N mm^-1^), although no significant differences (*p* ≥ 0.05) were observed between foliar and irrigation SA treatments (**Figure 1B**). The losses of firmness were significantly delayed (*p* < 0.05) by both SA treatments compared to untreated pepper fruits during postharvest storage. Thus, green pepper fruits treated with SA in preharvest had 1.19-fold in firmness values at 28 days of storage as compared with control (≈ 3.14 *vs.* 2.63 N mm^-1^, respectively). The increase was similar for both foliar application and irrigation at the end of storage (**Figure 1B**). The highest values of color, expressed in terms of hue°, were observed in those pepper fruits treated with foliar and irrigation SA at harvest (≈ 130 hue°), although no significant differences (*p* ≥ 0.05) were showed between both application methods (**Figure 1C**). On the other hand, color changes during postharvest storage were significantly delayed (*p* < 0.05) by both SA treatments and hue° values were higher in pepper fruits from SA treated plants (≈ 125 hue°) than in control (≈ 123 hue°) during the whole storage period, without significant differences between both SA treatments. It is well known, a higher hue° value showed a more intense dark green color in the pepper fruit (**Figure 1C**; **Supplementary Figure 5**).

Irrigation SA-treated green pepper fruits had a significantly higher (*p* < 0.05) content of total soluble solids (40.80 g kg^-1^) than control fruits at harvest (40.00 g kg^-1^), although those peppers treated with the foliar method did not show any significant differences (*p* ≥ 0.05) compared to untreated fruits (**Figure 1D**). Total soluble solids increased during postharvest storage at 7°C in all treatments, although the highest values were reached in foliar SA-treated fruits at 28 days of storage followed by those pepper fruits treated by irrigation methods (42.60 and 41.90 g kg^-1^, respectively). Thus, control pepper fruits showed the lowest values of total soluble solids of 41.10 g kg^-1^ at the end of postharvest storage at 7°C (**Figure 1D**). The highest levels of total acidity were observed in foliar SA treatment at harvest followed by irrigation SA treatment (1.56 and 1.44 g kg^-1^, respectively; **Figure 1E**). Total acidity decreased during postharvest storage until it reached the lowest values (0.93 g kg^-1^) in untreated fruits. However, those green pepper fruits treated with SA showed a content of total acidity around 1.12 g kg^-1^ at the end of storage, although no significant differences (*p* ≥ 0.05) were appreciated between both treatments. Therefore, both SA applied by foliar spraying and irrigation delayed losses of total acidity in green pepper fruit stored at 7°C for 28 days (**Figure 1E**). Ripening index was significantly higher (*p* < 0.05) in control pepper fruits at harvest (30.58) and after 28 days of postharvest storage (44.24) than SA treated ones (**Figure 1F**). When comparing both application methods studied for the SA application in preharvest, foliar SA treatment significantly showed (*p* < 0.05) the lowest ripening index with a value of 25.98 at harvest while the irrigation SA method was the most effective treatment to delay the ripening during postharvest storage (ripening index of 37.28; **Figure 1F**).

### 3.2 Foliar and irrigation SA enhances the bioactive compound content and the antioxidant capacity at harvest and during postharvest storage

**Figure 2:**
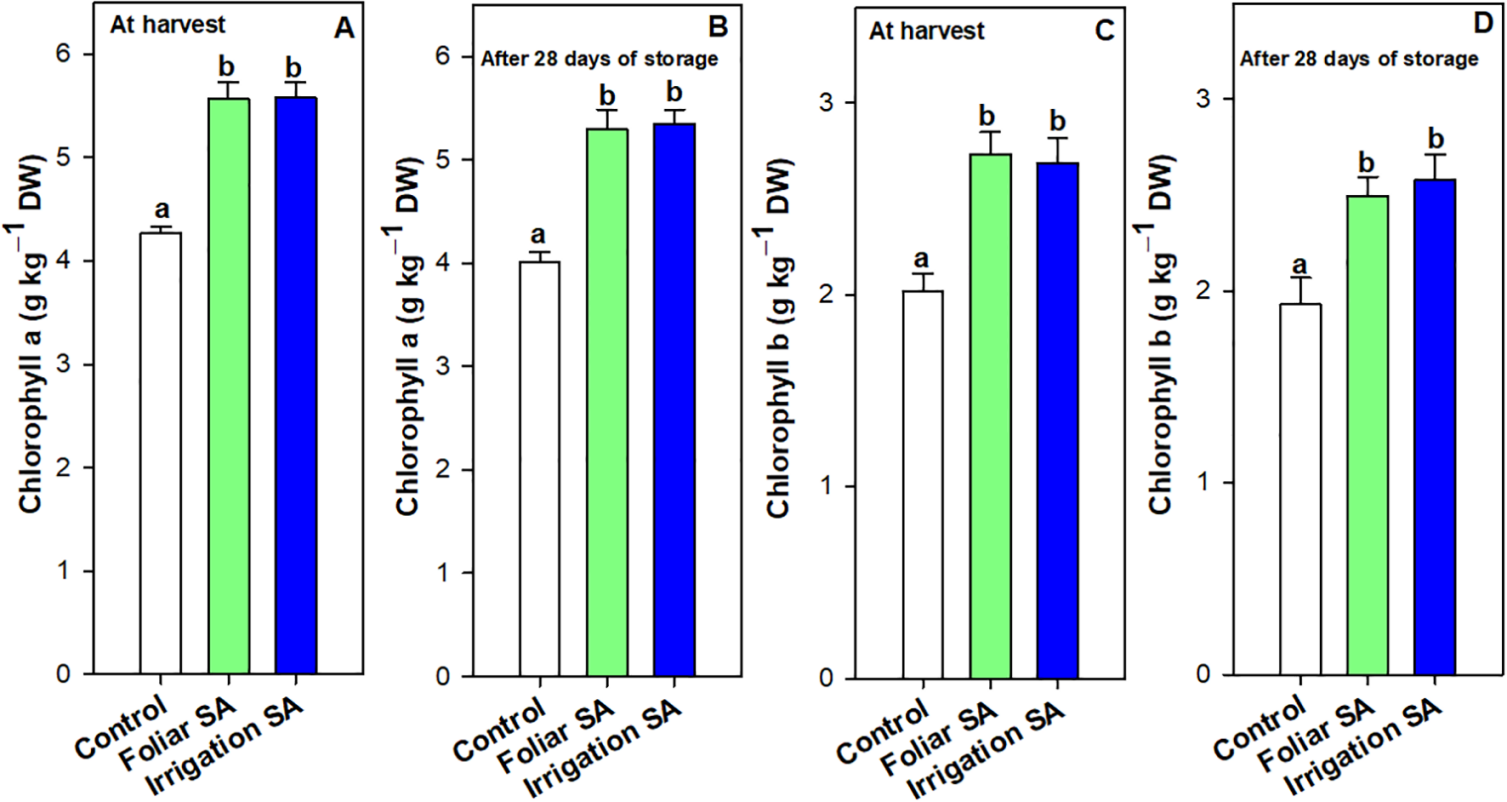
Effect of salicylic acid (SA) applied by foliar spraying [Foliar SA] and irrigation [Irrigation SA] on chlorophyll a and chlorophyll b content (g kg^-1^ DW) of green pepper fruit at harvest [**A, C**, respectively] and after 28 days of storage at 7°C [**B, D**, respectively]. Different lower case letters indicate significant differences at p < 0.05 according to Tukey’s HSD test among treatments for each parameter tested at harvest or after 28 days of storage..

The chlorophyll a and b content, as well as the total chlorophyll content calculated as the sum of both individual forms, followed the same pattern to all treatments for both studied times (**Figure 2**; **Supplementary Figure 2**). Chlorophyll a was the major chlorophyll pigment in green pepper fruit, ‘Herminio’ cv., since its content was 2-fold higher than chlorophyll b. Both SA treatments significantly increased (*p* < 0.05) the content of both chlorophylls than control at harvest (≈ 5.58 *vs*. 4.27 g kg^-1^ for chlorophyll a and ≈ 2.71 *vs.* 2.02 g kg^-1^for chlorophyll b) and after 28 days of storage (≈ 5.32 *vs.* 4.01 g kg^-1^ for chlorophyll a and 2.54 *vs.* 1.93 g kg^-1^ for chlorophyll b), although no significant differences were observed between foliar and irrigation SA treatments (*p* ≥ 0.05) (**Figures 2A–D**; **Supplementary Figures 2A, B**).

**Figure 3:**
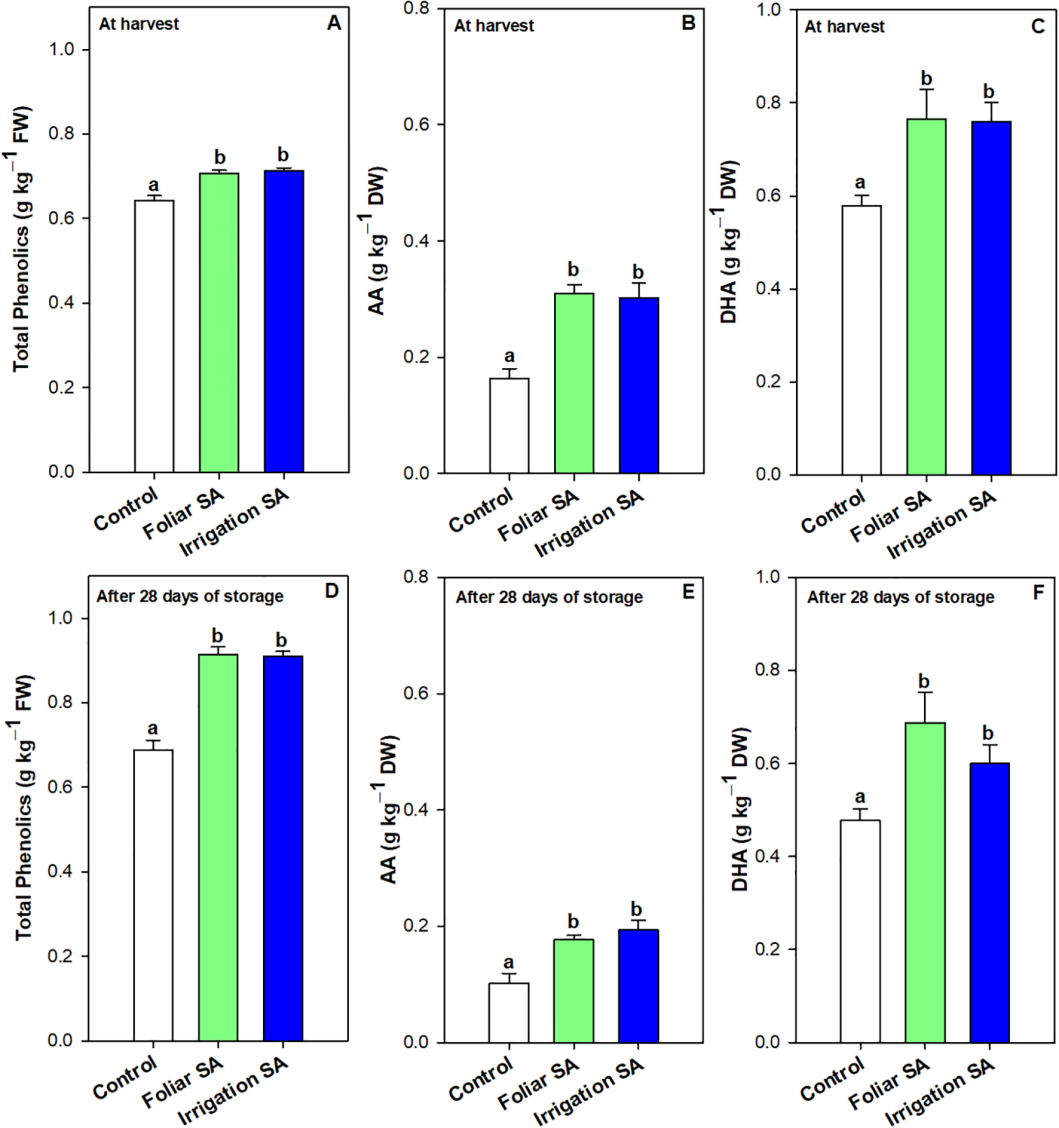
Effect of salicylic acid (SA) applied by foliar spraying [Foliar SA] and irrigation [Irrigation SA] on total phenolic (g kg^-1^ FW) and ascorbic acid (AA) and dehydroascorbic acid (DHA) content (g kg^-1^ DW) of green pepper fruit at harvest [**A–C**, respectively] and after 28 days of storage at 7°C [**D–F**, respectively]. Different lower case letters indicate significant differences at p < 0.05 according to Tukey’s HSD test among treatments for each parameter tested at harvest or after 28 days of storage.

Results showed that foliar and irrigation SA treatments reduced the rate of decline on chlorophyll a content (5.02 and 4.30%, respectively) after 28 days of storage compared to control (6.08%). However, only those green pepper fruits irrigated with SA showed the lowest rate of decline on chlorophyll b content. Thus, both SA treatments enhanced the total chlorophyll content in green pepper fruits at harvest (**Supplementary Figures 2A**) and after 28 days of storage (**Supplementary Figures 2B**) in the same way (*p* ≥ 0.05) than untreated fruits. This result could influence the maintenance of the intense dark green color of pepper fruit during postharvest, as it can be observed in **Supplementary Figure 5**. Total phenolic content was significantly enhanced (*p* < 0.05) at harvest and after 28 days of storage in those green pepper fruits treated with SA (0.71 and 0.91 g kg^-1^, respectively) compared to control fruits (0.64 and 0.69 g kg^-1^, respectively), although no significant differences (*p* ≥ 0.05) were appreciated between both application methods (**Figures 3A, D**). The significant differences between SA treated and untreated pepper fruits were higher at the end of the storage, showing an increase of 1.32-fold on total phenolics caused by SA treatment (**Figure 3D**). Compared with the values at harvest, the increase rate of phenolics compounds (21.97%) was enhanced by both SA applications during postharvest storage than control fruits (7.24%).

**Figure 4:**
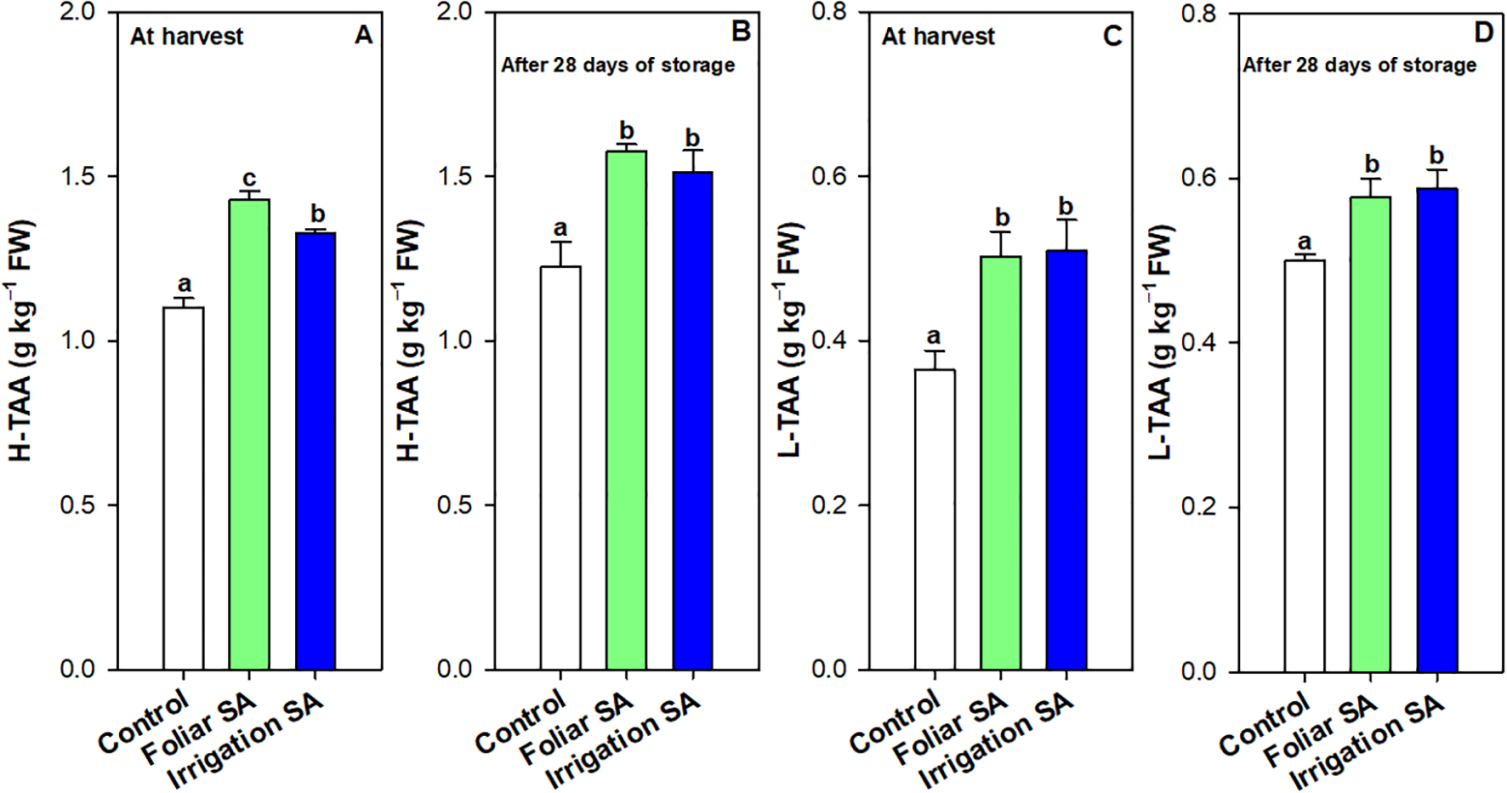
Effect of salicylic acid (SA) applied by foliar spraying [Foliar SA] and irrigation [Irrigation SA] on hydrophilic total antioxidant activity (H-TAA) and lipophilic total antioxidant activity (L-TAA) (g kg^-1^ FW) of green pepper fruit at harvest [**A, C**, respectively] and after 28 days of storage at 7°C [**B, D**, respectively]. Different lower case letters indicate significant differences at p < 0.05 according to Tukey’s HSD test among treatments for each parameter tested at harvest or after 28 days of storage.

Ascorbic acid (AA) and dehydroascorbic acid (DHA) content were enhanced with the SA preharvest application than control treatment both at harvest (≈ 0.30 *vs.* 0.16 g kg^-1^ for AA and 0.76 *vs.* 0.58 g kg^-1^ for DHA) and after 28 days of storage (≈ 0.19 *vs.* 0.10 g kg^-1^ for AA and 0.65 *vs.* 0.48 g kg^-1^ for DHA). Nevertheless, no significant differences (*p* ≥ 0.05) were observed between both SA application ways. The DHA content was 2-fold higher than AA content in green pepper fruit and both forms of vitamin C were degraded during storage for all treatments. However, SA treatment delayed this functional degradation by 44% (**Figures 3B, C, E, F**). In fact, the irrigation SA treatment showed a decrease rate on AA content of 36.66% than control green pepper fruits (37.50%) from harvest until 28 days of storage at 7°C. Nevertheless, those pepper fruits harvested from plants treated with SA by foliar spraying presented the lowest percentage of decrement on DHA content (9.21%) compared with the control ones (17.24%). Total vitamin C, expressed as the sum of both AA and DHA forms, was also significantly enhanced (*p* < 0.05) by the two SA treatments studied in the same proportion (**Supplementary Figures 3A, B**). Hydrophilic (H-TAA) and lipophilic (L-TAA) total antioxidant activity was significantly improved (*p* < 0.05) with SA preharvest treatments compared to control both at harvest (≈ 1.38 *vs.* 1.10 g kg^-1^ for H-TAA and 0.51 *vs.* 0.36 g kg^-1^ for L-TAA) and after 28 days of storage (≈ 1.55 *vs.* 1.23 g kg^-1^ for H-TAA and 0.59 *vs.* 0.50 g kg^-1^ for L-TAA) (**Figures 4A–D**). Specifically, foliar SA application was the most effective treatment stimulating the H-TAA at harvest compared to other treatments (**Figure 4A**). However, these significant differences (*p* < 0.05) between both application methods of SA were not observed after 28 storage days (**Figure 4B**). The increment of H-TAA from harvest to the end of the postharvest period was highest in those green pepper fruits irrigated with SA compared to control treatment (11.92 *vs.* 9.83%). Similarly, no significant differences (*p* ≥ 0.05) were appreciated between foliar and irrigation SA methods on L-TAA (**Figures 4C, D**). Finally, carotenoids content did not show any significant differences (*p* ≥ 0.05) among treatments (**Supplementary Figures 4A, B**).

### 3.3 Foliar and irrigation SA modulates antioxidant enzyme activities and the relative antioxidant systems-based gene expression at harvest and during postharvest storage

**Figure 5:**
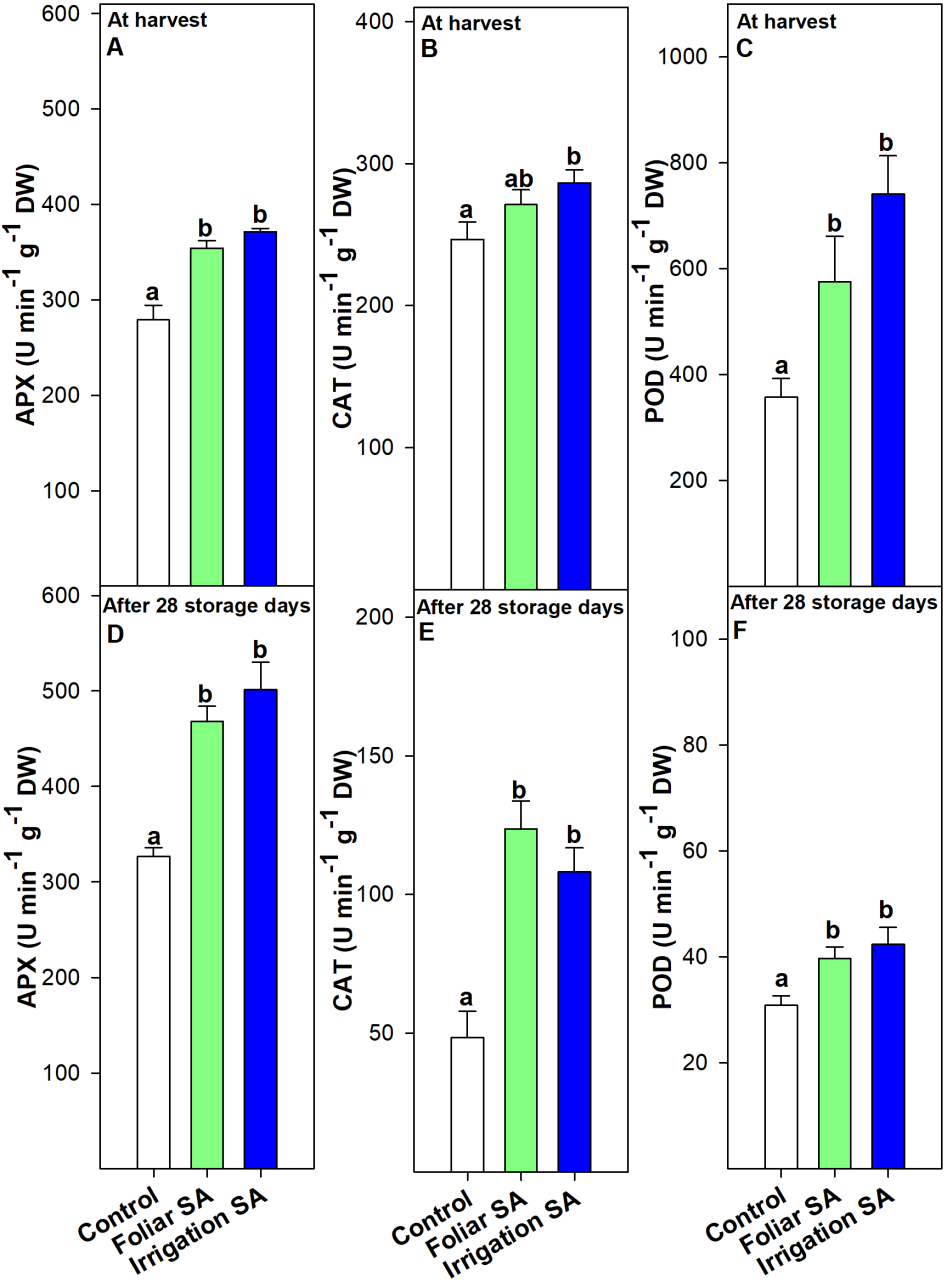
Effect of salicylic acid (SA) applied by foliar spraying [Foliar SA] and irrigation [Irrigation SA] on ascorbate peroxidase (APX), catalase (CAT) and peroxidase (POD) activities (U min^-1^ g^-1^ DW) of green pepper fruit at harvest [**A–C**, respectively] and after 28 days of storage at 7°C [**D–F**, respectively]. Different lower case letters indicate significant differences at p < 0.05 according to Tukey’s HSD test among treatments for each parameter tested at harvest or after 28 days of storage.

**Figure 6:**
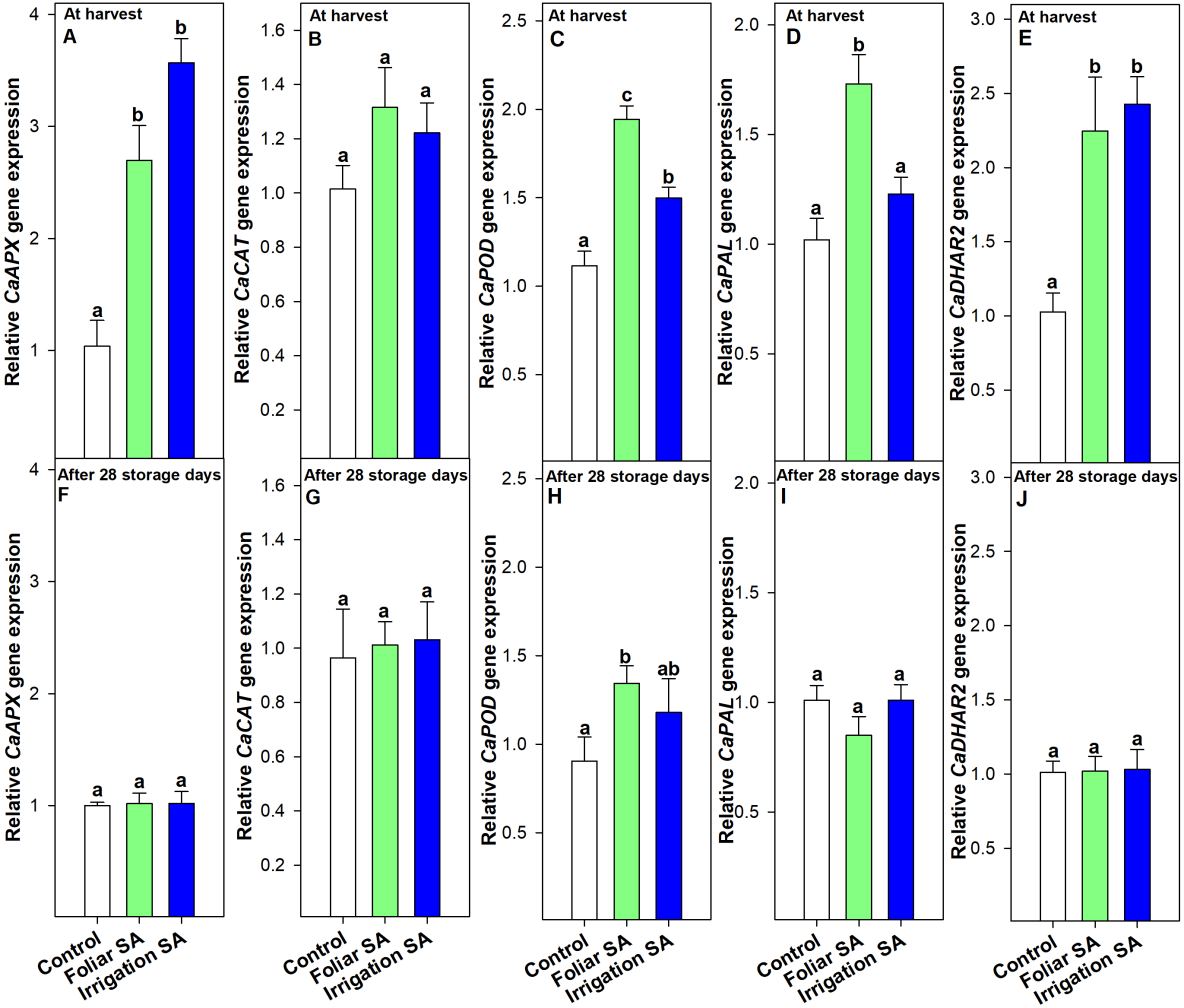
Effect of salicylic acid (SA) applied by foliar spraying [Foliar SA] and irrigation [Irrigation SA] on the relative *CaAPX*, *CaCAT*, *CaPOD*, *CaPAL*, and *CaDHAR2* genes expression of green pepper fruit at harvest [**A–E**, respectively] and after 28 days of storage at 7°C [**F–J**, respectively]. Different lower case letters indicate significant differences at p < 0.05 according to Tukey’s HSD test among treatments for each parameter tested at harvest or after 28 days of storage.

Foliar and irrigation SA treatments significantly stimulated (*p* < 0.05) the APX activity compared to control at harvest (≈ 354 and 372 U min^-1^ g^-1^, respectively, *vs.* 279 U min^-1^ g^-1^) and after 28 days of storage (≈ 468 and 501 U min^-1^ g^-1^, respectively, *vs.* 327 U min^-1^ g^-1^), although no significant differences (*p* ≥ 0.05) were appreciated between both application methods (**Figures 5A, D**). This effect could be related to the upregulation of the relative *CaAPX* gene expression detected by foliar and irrigation SA treatments at harvest (relative expression of 2.70 and 3.57, respectively), disappearing this effect after 28 days of storage at 7°C (**Figures 6A, F**).

CAT activity was only significantly stimulated (*p* < 0.05) in those green pepper fruits treated with SA by irrigation at harvest (≈ 286 U min^-1^ g^-1^) and no significant differences (*p* ≥ 0.05) were observed between foliar SA and control treatments (≈ 271 and 247 U min^-1^ g^-1^, respectively) (**Figure 5B**). Nevertheless, this difference was accentuated during postharvest storage and both SA preharvest treatments significantly increased (*p* < 0.05) the activity of CAT antioxidant enzyme than control fruits in the same way (≈ 116 *vs.* 48 U min^-1^ g^-1^) (**Figure 5E**). When the effect of SA was studied on the relative *CaCAT* gene expression, no significant differences (*p* ≥ 0.05) were observed on the modulation of this targeted gene either at harvest nor during postharvest storage (**Figures 6B, G**). Foliar and irrigation SA treatment significantly activated (*p* < 0.05) the POD enzyme at harvest reaching values of 575.46 and 741.02 U min^-1^ g^-1^, respectively, compared with control (356.75 U min^-1^ g^-1^) (**Figure 5C**). This effect was also observed after 28 days of storage since those foliar and irrigation SA-treated green pepper fruits exhibited values of POD of 39.65 and 42.34 U min^-1^ g^-1^, respectively, compared with untreated fruits (30.85 U min^-1^ g^-1^) (**Figure 5F**). In this sense, SA applied by foliar spraying and irrigation significantly upregulated (*p* < 0.05) the relative *CaPOD* gene expression at harvest by 1.94 and 1.50, respectively (**Figure 6C**). Thus, the highest effect was observed for the preharvest foliar treatment, and it was maintained after 28 storage days only in those green pepper fruits treated with foliar SA with a relative expression of 1.34 (**Figure 6H**). The relative *CaPAL* gene expression was only upregulated at harvest with a value of 1.73 after the foliar SA application, while both foliar and irrigation SA treatments upregulated the relative *CaDHAR2* gene expression at harvest in green pepper fruits (relative expression of 2.25 and 2.43, respectively) (**Figure 6E**). However, SA did not modulate (*p* ≥ 0.05) the expression of these two targeted genes after 28 days of storage at 7°C (**Figures 6I, J**).

## 4 Discussion

Bell pepper (*Capsicum annuum* L.) is a model for studying the ripening and senescence processes of non-climacteric fleshy fruit, during which numerous physiological changes occur, the most noticeable being the color change caused by chlorophyll degradation and the synthesis of new pigments such as carotenoids (Chaki et al., 2015; Ma et al., 2021). Nevertheless, numerous changes occur during the ripening of bell pepper fruit, including alterations in flavor, aroma, and texture, which are regulated by both external and internal factors (Palma et al., 2011; Klie et al., 2014). For instance, phenological stages and harvest dates are two key factors that significantly influence some nutritional and functional traits of green pepper fruit, as it was unveiled by Dobón-Suárez et al. (2021b). Fruit ripening and senescence involve complex and highly coordinated molecular and biochemical processes that include ripening-associated genes, transcription factors, enzymes, repressors, signaling molecules, and metabolic pathways in both climacteric and non-climacteric fruits (Cherian et al., 2014; Fuentes et al., 2019). These processes influence fruit quality on one hand and postharvest losses on the other.

**Figure 7:**
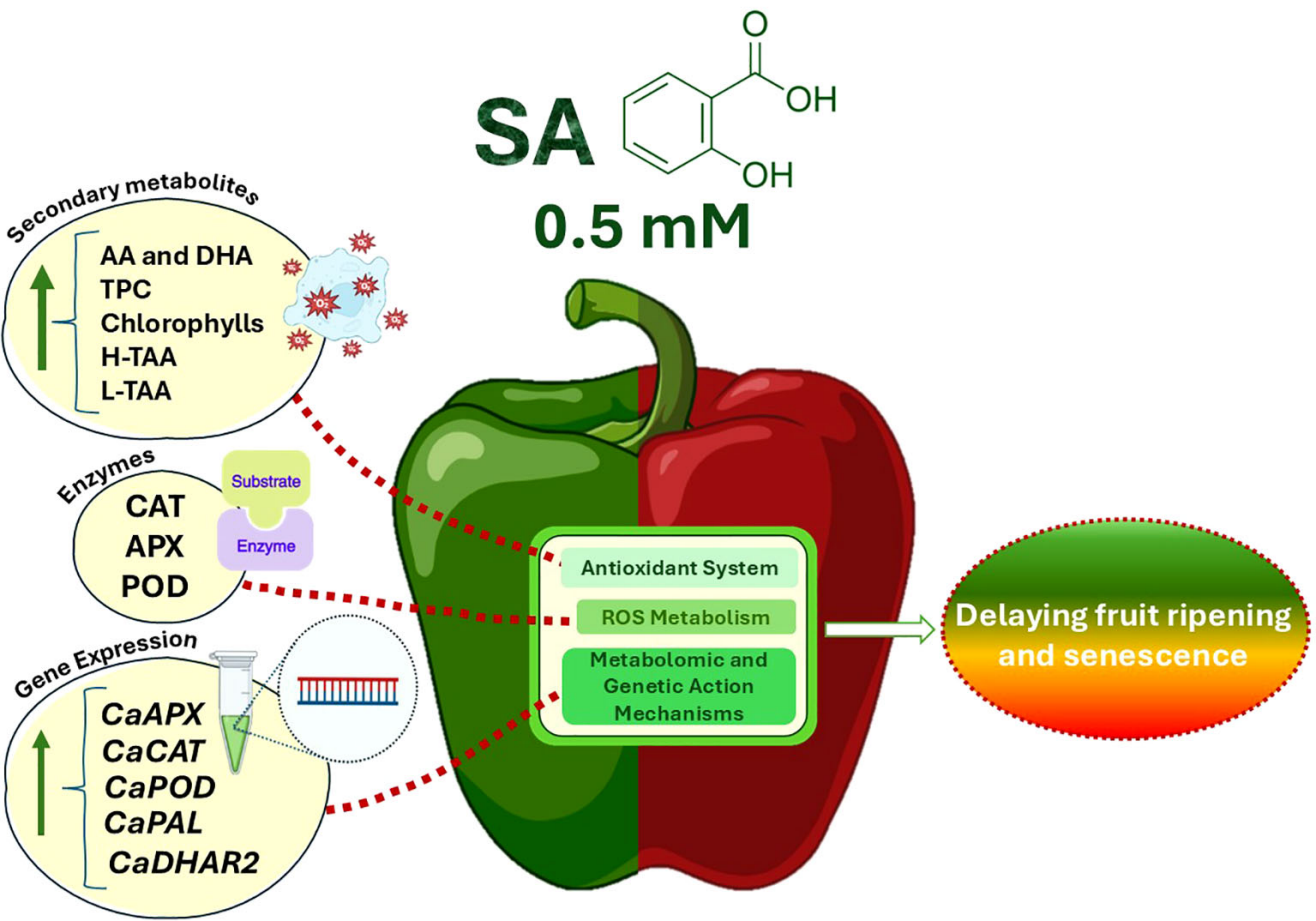
A hypothetical working model illustrates the role of preharvest 0.5 mM SA treatment delaying green pepper fruit ripening and senescence during storage at 7°C using a regulatory network model for some targeted metabolites and genes. Green arrows represent stimulation of metabolites or functional parameters and an up-regulation of the gene expression. SA preharvest treatment at 0.5 mM increases secondary metabolites (ascorbic acid and dehydroascorbic acid, total phenolic content, carotenoids and chlorophylls) and stimulates both the hydrophilic and lipophilic total antioxidant activity, as well as antioxidant enzymes (catalase, ascorbate peroxidase and peroxidase), reducing probably ROS accumulation, thereby delaying fruit ripening and senescence. This metabolomic modulation has been mediated by increasing the expression of *CaAPX*, *CaCAT*, *CaPOD*, *CaPAL* and *CaDHAR2* genes by SA [For interpretation of the references to color in this figure legend, the reader is referred to the web version of this article]. Abbreviations: SA (salicylic acid), AA (ascorbic acid), DHA (dehydroascorbic acid), TPC (total phenolic content), H-TAA (hydrophilic-total antioxidant activity), L-TAA (lipophilic-total antioxidant activity), CAT (catalase), APX (ascorbate peroxidase), POD (peroxidase), *CaAPX* [*L*-ascorbate peroxidase (APX) gene], *CaCAT* [catalase (CAT) gene], *CaPOD* [peroxidase (POD) gene], *CaPAL* [phenylalanine ammonia-lyase (PAL) gene] and *CaDHAR2* [dehydroascorbate reductase 2 gene], ROS (Reactive Oxygen Species).

As fruit ripens or undergoes senescence, it becomes more susceptible to fungal pathogens (Alkan and Fortes, 2015), leading to green pepper fruit deterioration. Cold storage is widely adopted to prevent premature ripening and senescence since this fruit is highly perishable at ambient temperatures. However, green bell pepper is susceptible to chilling injury (CI) at temperatures below 7°C (Lim et al., 2007). Consequently, common strategies to slow down senescence and preserve fruit quality include both pre- and postharvest management practices and technological tools. Many factors, however, such as various plant hormones and biotic and abiotic stresses are known to influence bell pepper fruit ripening (Sun et al., 2015; Cheng et al., 2016). Recently, numerous studies have shown that salicylic acid (SA) preharvest treatment applied by foliar spraying influences the ripening and senescence of fruit species, such as sweet cherry (Giménez et al., 2014), table grape (Champa et al., 2014; Gomes et al., 2021), jujube fruit (Shanbehpour et al., 2020), pomegranate fruit (García-Pastor et al., 2020), lemon (Serna-Escolano et al., 2021) and green pepper (Dobón-Suárez et al., 2021a), showing activation of the antioxidant system and a delay in fruit senescence, as it was highlighted by Chen S. et al. (2023). The present study showed that SA preharvest treatment applied by both foliar spraying and irrigation enhanced the fruit quality of green pepper fruit resulting in a slowdown of the ripening and senescence processes during postharvest storage at 7°C (**Supplementary Figure 5**). As it can be observed in **Figure 7**, this fact could be modulated throughout the stimulation of the antioxidant system accompanied by an increase on the content of secondary metabolites and the activity of antioxidant enzymes that is mediated by the upregulation of their codifying gene expressions.

**Figure 8:**
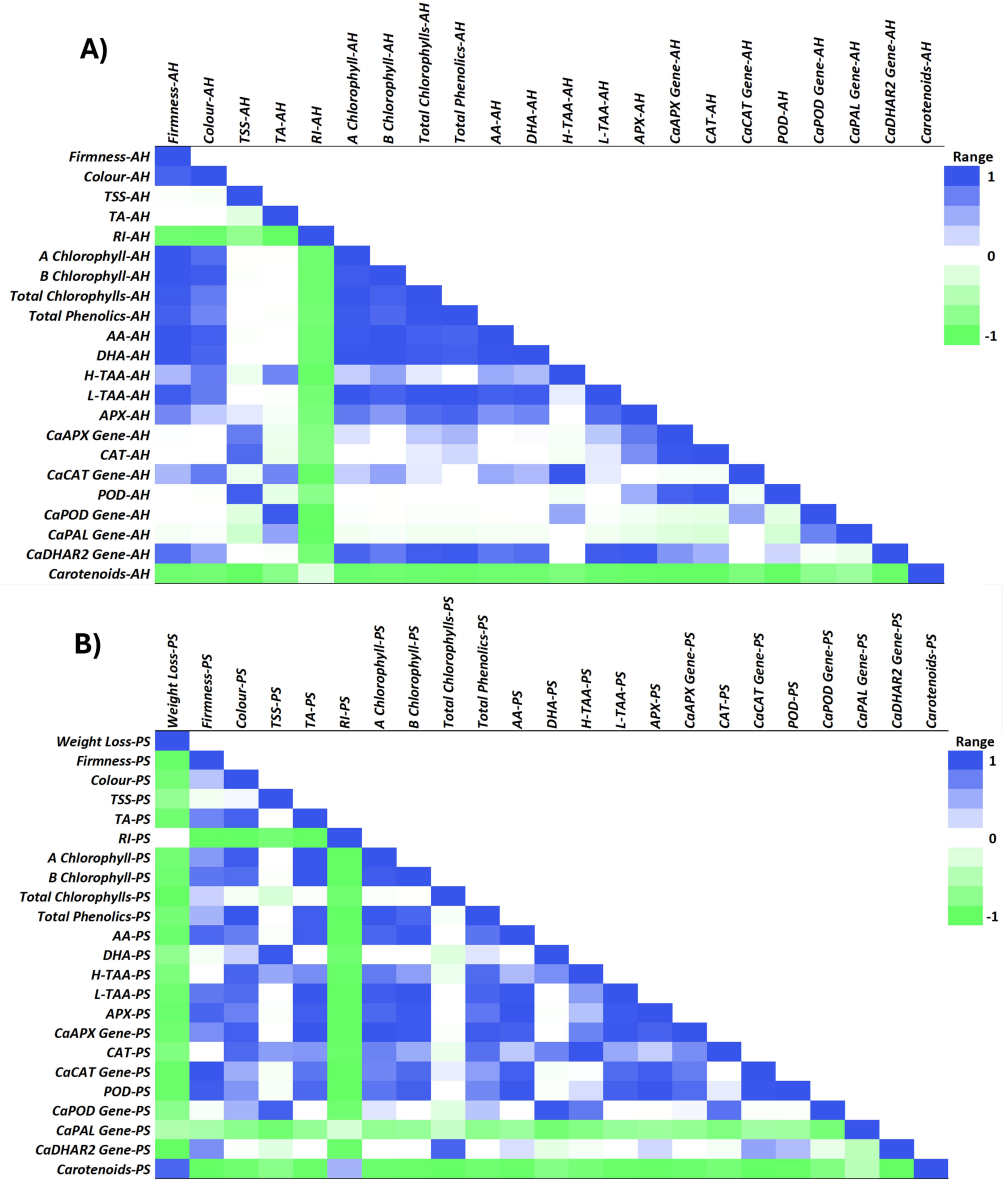
Pearson correlation heatmap of green pepper fruit qualities, metabolites and antioxidant system and targeted genes at harvest (AH) **(A)** and after 28 days of postharvest storage (PS) at 7°C **(B)**. Fruit quality includes weight loss, firmness, color, total soluble solids (TSS), total acidity (TA) and ripening index (RI). Metabolites and antioxidant systems include chlorophyll a, chlorophyll b, total chlorophylls, total phenolics, ascorbic acid (AA), dehydroascorbic acid (DHA), hydrophilic-total antioxidant activity (H-TAA), lipophilic-total antioxidant activity (L-TAA), ascorbate peroxidase (APX), catalase (CAT), peroxidase (POD), and carotenoids. Targeted genes based on the antioxidant enzymatic system including the relative expression of *CaAPX* gene, *CaCAT* gene, *CaPOD* gene, *CaPAL* gene and *CaDHAR2* gene. The range runs from -1 = green to 1 = blue, which represents the correlation coefficient between the fruit qualities and metabolomic and genetic parameters of the antioxidant system run from -1 to 1.

Fruit quality is related to the ripening and senescence of fruit during storage, which is involved in fruit softening, weight loss, color change, ethylene production and respiration rate (Gao et al., 2020). Salicylic acid (SA) regulates growth in plants, playing an efficient role in growth and development, flowering and fruit ripening, and photosynthesis (Natasha et al., 2020). Many studies have also indicated that SA and its derivatives play an important role in regulating the physiological metabolism of fruit to achieve optimal fruit quality and to maintain it during postharvest (Valverde et al., 2015; Giménez et al., 2016; Hanif et al., 2020; Amiri et al., 2021; Chen S. et al., 2023). Over the past ten years, biochemical data have also indicated that the bell pepper fruit ripening process is influenced by the metabolism of reactive oxygen species (ROS) and nitrogen oxygen species (NOS) (Palma et al., 2015; Corpas and Palma, 2018; Corpas et al., 2018), which reflects the profound biochemical and molecular changes taking place during ripening (Camejo et al., 2015). The accumulation of the reactive oxygen species (ROS) including hydroxyl radical, superoxide and hydrogen peroxide during fruit ripening can cause oxidative damage leading to membrane lipid breakdown and loss of cellular turgor, triggering cell death and damage in fruit tissue (Champa et al., 2014). The beneficial effect of SA has been recently related to its capacity to improve photosynthesis and the activity of antioxidant enzymes, leading to maintaining the balance between the production and elimination of ROS (Batista et al., 2019). In the present study, results show a significant effect of both foliar and irrigation SA on activating the APX and POD by upregulating the relative *CaAPX* and *CaPOD* gene expression, respectively, at harvest (**Figures 5A, C**, **6A, C**). Results showed that SA treatment applied by foliar spraying was more effective on increasing *CaPOD* gene expression than SA, irrigation treatment, although this effect was not reflected in a higher POD activity in those foliar SA-treated peppers than the irrigated ones (**Figure 5C**). Nevertheless, SA treatment applied by irrigation was the most effective in stimulating CAT activity at harvest (**Figure 5B**), although this effect was not mediated through the upregulation of the relative *CaCAT* gene expression (**Figure 6B**). The effect of SA treatment on activating the antioxidant enzymes activity at harvest has been demonstrated in the present study and results show that this plant growth regulator could have a potential effect modulating the antioxidant enzymes-gene expression. These results propose that the enzymatic antioxidants can offset the damaging effects of ROS metabolism on cell structure (Gomes et al., 2021; Serna-Escolano et al., 2021), which results in a slowdown in ripening process and an increase on quality traits at harvest (**Figure 1**). In fact, the ripening index (RI) was deeply correlated (negatively, Pearson) with firmness, color, TSS, TA, chlorophylls, phenolics, antioxidant capacity and antioxidant enzymatic system, except with the carotenoids content which also showed a similar correlation pattern (**Figure 8A**). Some studies corroborate these findings regarding the activation of enzymatic systems by the application of SA and its derivatives [Methyl salicylate (MeSA) and Acetylsalicylic acid (ASA)], leading to the high antioxidant activity in sweet cherry (Giménez et al., 2014; Zhang et al., 2011), pomegranate fruit (García-Pastor et al., 2020), citrus (Zhu et al., 2016), papaya (Hanif et al., 2020) and banana (Xu et al., 2019).

Phenolic compounds have antioxidant activity, which not only scavenge free radicals and reduce oxidative damage to fruits, but also contribute to fruit flavor and quality maintenance. Their biosynthesis is involved in phenylpropanoid pathway of plant secondary metabolites. Phenylalanine ammonia-lyase (PAL) is the first step to catalyze the conversion of phenylalanine to cinnamic acid which is further converted to phenolic acid (Oraei et al., 2019). The enzyme activity of PAL could be enhanced by SA (Zhou et al., 2018), increasing phenolic content in oranges (Amiri et al., 2021) and table grapes (Blanch et al., 2020). In the present study, SA treatment applied by foliar spraying upregulated the relative *CaPAL* gene expression at harvest (**Figure 6D**), which led to a significant increase in the total phenolic content (**Figure 3A**). However, the irrigation-SA treatment also showed an enhancement in phenolic compounds as compared with control (**Figure 3A**), although these findings were not corroborated by the analysis of the *CaPAL* targeted gene (**Figure 6D**). In this sense, previous transcriptomic and metabolic profiling of watermelon uncovered the role of SA pretreatment up-regulating the expression of flavonoid biosynthesis genes, thus increasing the total flavonoid content (Liu et al., 2023). Furthermore, transcriptome analysis and exogenous SA treatment demonstrated that SA (NPR1) is involved in the positive regulation of flavonoid biosynthesis (Wu et al., 2021).

On the other hand, SA treatment increased ascorbic acid content which is an essential plant antioxidant vital for defense against oxidative stress, leading to alleviating damages induced by ROS accumulation. In this sense, the content of both AA and DHA was quantified at harvest (**Figures 3B, C**), as well as the relative *CaDHAR2* gene expression which is involved in the biosynthesis of the cytoplasmic enzyme DHAR2, namely dehydroascorbate reductase, implicated on the catalyzation of the glutathione (GSH)-dependent reduction of dehydroascorbate and had a direct role in regenerating ascorbic acid (**Figure 6E**). Results suggest that both SA applications (foliar and irrigation) effectively enhanced the AA and DHA content, and consequently, the total vitamin C content, throughout the upregulation of the *CaDHAR2* gene expression at harvest, although no significant differences were appreciated between both methods (**Figures 6E**, **3B, C**; **Supplementary Figure 3A**). The form of AA can neutralize radicals to retard oxidative reactions triggering the ripening process in plant tissues (Huang et al., 2017; Sangprayoon et al., 2019). Other studies corroborate these findings hypothesizing that the application of SA could enhance the antioxidant ability by inhibiting the ascorbic acid oxidase (AAO) enzyme, which would affect the ascorbate-glutathione cycle positively, leading to the high content of AA in orange fruit (Amiri et al., 2021; Hanif et al., 2020; Wei et al., 2011). Other reports indicated that SA, MeSA, or ASA treatments could lead to high content of DHA in fruit, which is oxidized from AA by the AAO enzyme in pomegranate fruit and table grapes (García-Pastor et al., 2020; Hazarika and Marak, 2022).

Bell pepper ripening is characterized by important visual and metabolic changes regulated by transcription factors, with color changes caused by chlorophyll degradation and biosynthesis of new pigments such as carotenoids (Chaki et al., 2015). In the present study, both SA treatments applied by foliar spraying and irrigation significantly influenced the content of these pigments at harvest (**Figures 2A, C**; **Supplementary Figure 2A**), the highest levels of chlorophylls a and b were recorded in those green pepper fruits harvested from 0.5 mM SA-treated plants (**Supplementary Figure 5**). Multiple biological functions have been reported for chlorophylls as lipophilic-nature pigments. Strictly related to their antioxidant capabilities, two main mechanisms can be described: Their direct free-radical-scavenging activity and the metabolic activation of detoxification pathways, as was reported by Pérez-Gálvez et al. (2020). Accordingly, exogenous SA increased chlorophyll content under drought and salinity conditions (Tang et al., 2017; Ghassemi-Golezani et al., 2018). Some studies showed that there was a positive correlation between TPC and TAA (Amiri et al., 2021; Wei et al., 2011), considering also the contribution of ascorbic acid. The present study shows the positive correlation observed between total phenolics and AA and DHA content (**Figure 8A**). In this sense, the H-TAA (**Figure 4A**) was significantly improved by SA preharvest treatments at harvest, although the highest stimulation was achieved with foliar spraying. Since no significant differences were recorded between the two application methodologies on total phenolics or vitamin C content (**Figures 3A–C**; **Supplementary Figure 3A**), probably other hydrophilic antioxidant compounds, such as glutathione (GSH), could be highly influenced by foliar SA treatment. As opposed to H-TAA, the analyses of the L-TAA at harvest showed that this parameter was stimulated by both SA applications, although no significant differences were observed between both methodologies (**Figure 4C**). This functional increment with the application of SA at 0.5 mM is corroborated by a previous study in ‘Lamuyo’ green pepper fruit and could be related to the enhancement chlorophyll a and b content by the application of SA that has been demonstrated in the present study (**Figures 2A, C**; **Supplementary Figures 2A**, **5**). Preharvest applications of SA have been reported to increase the antioxidant capacities of grapes (Champa et al., 2014; Gomes et al., 2021), Indian jujube (Shanbehpour et al., 2020), lemon (Serna-Escolano et al., 2021), pomegranate fruit (García-Pastor et al., 2020), and sweet cherry (Giménez et al., 2014). Accordingly, SA preharvest treatment has a beneficial impact on the quality of green pepper fruit, ‘Herminio’ cv., at harvest which results in a slowdown in the ripening process (**Figure 1**; **Supplementary Figure 5**). At harvest, quality traits such as color and firmness were positively correlated and both showed a deep correlation (positively, Pearson) with the antioxidant compounds and the activity of the antioxidant enzymes, although no correlation was observed with the targeted genes (**Figure 8A**). Specifically, the relative expression of *CaDHAR2* gene was highly correlated with firmness, color, chlorophylls, phenolics, AA, DHA, L-TAA, APX and the expression of *CaAPX* gene (**Figure 8A**; positively, Pearson). The action mechanism proposed in the present study is the following: *1)* SA induces the synthesis of secondary metabolites and enhances the antioxidant systems by stimulating the phenylpropanoid biosynthesis pathway, and *2)* SA activates the antioxidant enzymes, acting on the modulation of the relative antioxidant systems-based genes expression and regulating the balance between the antioxidant system and ROS metabolism, contributing to antioxidant metabolism insights (**Figure 7**).

The senescence delay triggered by the foliar and irrigation application of 0.5 mM SA extended the shelf-life of ‘Lamuyo’ green pepper fruit since the preharvest treatment reduced fruit quality losses (**Figure 1**; **Supplementary Figure 5**). The contribution to extending shelf-life and delaying fruit senescence for 28 days at 7°C could be attributed to the enhancement of both total phenolics (**Figure 3D**) and antioxidant capacity from hydrophilic and lipophilic phases (**Figures 4B, D**) with both SA applications which might reduce the oxidative damage (**Figure 7**). Regarding the functional increment related to phenolics content after harvest, results showed the highest increase rate in those green pepper harvested from SA-treated plants. However, the highest increment on H-TAA after 28 storage days was observed in those pepper fruits irrigated with SA, leading to a higher stimulating effect of the antioxidant system in those pepper fruits treated with SA. Similar findings applying salicylates in preharvest have been obtained in sweet cherry (Valverde et al., 2015; Zhang et al., 2011), pomegranate fruit (García-Pastor et al., 2020), lemon (Serna-Escolano et al., 2021) and plum (Davarynejad et al., 2015). Other contributing aspects to the delay in senescence could be related to the higher levels of two forms of ascorbic acids (AA and DHA; **Figures 3E, F**) quantified in those green pepper fruits treated with foliar and irrigation SA. Moreover, foliar and irrigation SA treatments lead to higher increase rate on DHA and AA content, respectively, compared to control from harvest until 28 days of storage. In this sense, Ruoyi et al. (2005) indicated that this effect is mediated by the inactivation of the AAO enzymatic activity, which might have caused a delay in the senescence of peppers in the later stages of the storage period. The inhibition of AAO was also advantageous in keeping vitamin C and for anti-browning in sweet pepper (Rao et al., 2011). Recently, the coordinated regulatory network of ncRNAs involved in the ripening of bell pepper fruit has been analyzed (Zuo et al., 2019), providing a theoretical basis for deciphering novel mechanisms of fruit ripening in future studies. In the present study, the increase in secondary metabolites, such as phenolics or vitamin C, mediated by SA was not correlated with the upregulation of the relative *Ca*PAL gene expression after 28 days of storage at 7°C, as it can be observed in **Figure 8B**. Similarly, both SA applications did not modulate the response of *CaDHAR2* gene after 28 storage days (**Figure 6J**). This finding could be related to the fact that SA was applied in preharvest upregulating both genes at harvest, and probably during the crop cycle, although the modulation effect is lost during postharvest storage.

On the other hand, a delay in senescence is commonly associated with a delay in color changes and this effect was observed in the present study from a metabolomic approach, where chlorophylls a and b content were significantly higher in both foliar and irrigation SA-treated pepper fruits than control (**Figures 2B, D**; **Supplementary Figure 2B**), as it was previously discussed. In addition, the rate of decline on chlorophyll a and b from harvest date until the end of postharvest storage was lower in both SA treatments, especially with the irrigation method, compared to control. This result shows a preserving effect on green color maintenance after postharvest storage associated with the SA treatments, as it can be observed in **Supplementary Figure 5**. After 28 days of storage at 7°C, a similar effect was observed on the relative expression of *CaAPX* and *CaCAT* genes which was no upregulated by SA treatments (**Figures 6F, G**), although an enzymatic activity stimulation was observed compared to untreated pepper fruit because of both foliar and irrigation SA treatments (**Figures 5D, E**). Nevertheless, SA treatment applied by foliar spraying positively modulated the relative expression of *CaPOD* gene after 28 storage days and, therefore, stimulated the activity of the POD enzyme (**Figures 5F**, **6H**). Induction POD activity, which is an important oxyradical detoxification enzyme in plant tissues, has been demonstrated in the present study and may generally facilitate conditions that can delay senescence in green peppers fruits (Zhou et al., 2011). Furthermore, the only relative expression of those antioxidant enzyme activities-related genes that was upregulated after 28 days of storage was for the POD enzyme (**Figure 6H**). These results are in accordance with those reported by Rao et al. (2011) in which 1- and 2-mM SA postharvest treatments extended the shelf-life of sweet pepper fruit (*Capsicum annum* L., cv. Indra). Senescence is associated with the defensive system, including antioxidant enzymes, such as POD. In the present study, POD was highly correlated (positively, Pearson) with firmness, color, TA, chlorophylls, phenolics and AA content, L-TAA, APX and the relative expression of both *CaAPX* and *CaCAT* genes (**Figure 8B**). An efficient antioxidant system can postpone the senescence process even though antioxidative activity in fruits decreases with ageing (Zheng et al., 2007). Antioxidants can delay, retard or prevent oxidation processes by reacting with free radicals, chelating metals and acting as oxygen scavengers, a triplet as well as singlet form and transferring hydrogen atoms to the free radical structure. Similar results were recently reported where the incorporation of SA foliar spraying and caraway oil coating resulted in the highest antioxidant enzyme activity and the lowest chilling injury in treated pepper fruits stored under cold conditions (Hanaei et al., 2022). The results of the present study reinforce the knowledge gap about the effect of SA applied by foliar spraying and irrigation on the complex interaction of metabolites and genes in regulating the bell pepper fruit ripening and senescence processes.

Finally, whilst not the primary focus of this study, it has been demonstrated that the foliar and irrigation application of SA on bell pepper plants has the capacity to enhance fruit quality without exerting a detrimental effect on productivity. The results suggest that both foliar and irrigation SA treatments improve the accumulated crop yield, expressed in terms of kg per plant, throughout the crop cycle of ‘Lamuyo’ green pepper fruit, ‘Herminio’ cv., as it can be observed in **Supplementary Figure 1**. However, no significant differences were appreciated between the two application methodologies (*p* ≥ 0.05). The role of SA in improving fruit yield may have been due to the translocation of more photoassimilates to fruits, thereby increasing fruit weight and reducing negative environmental impacts. These findings confirm those observed in a previous study where an increase of 0.5 kg yield was recorded in the last harvest date after the foliar application of SA (Dobón-Suárez et al., 2021a). Other studies also observed a higher productivity with the preharvest application of exogenous SA in different types of pepper plants (Sobczak et al., 2023; Preet et al., 2023; Ghahremani et al., 2023; Ibrahim et al., 2019; Elwan and El-Hamahmy, 2009).

In summary, the results of the present study demonstrated that both SA preharvest methods tested (foliar and irrigation) were effective in enhancing crop yield, fruit quality at harvest, and delaying ripening and senescence of green pepper fruit throughout the antioxidant metabolism. The effect of both SA applications was found to be similar in most of the parameters analyzed, although it should be highlighted that they showed differences as follows: Firstly, foliar spraying of SA led to a positive modulation of the relative expression of *CaPOD* and *CaPAL* genes at harvest, although this stimulation was only upregulated for the *CaPOD* gene after 28 days of storage. Secondly, irrigation with SA led to an enhancement of H-TAA at harvest and TSS content after postharvest storage. Thirdly, the irrigation method of SA showed a significant increase in CAT activity and delayed weight loss and ripening index. However, regarding their impact on the field and on the agri-food industry, it should be emphasized that the irrigation method was the most beneficial due to the ease and cost-effectiveness with which it could be applied in comparison to foliar spraying.

## 5 Conclusions

In our study, we found that salicylic acid (SA) preharvest treatment, both by foliar spraying and irrigation, improved the fruit quality of green peppers, resulting in a slowing of the ripening and senescence processes during postharvest storage, while stimulating the antioxidant system, accompanied by an increase in the content of secondary metabolites and activity of antioxidant enzymes, mediated by the upregulation of the relative response of genes responsible for their biosynthesis. The mechanism of action proposed in the present study is as follows: *1)* SA induces the synthesis of secondary metabolites and enhances the antioxidant systems by stimulating the phenylpropanoid biosynthesis pathway, and *2)* SA activates the antioxidant enzymes by acting on the modulation of the relative expression of antioxidant system-based genes and regulating the balance between the antioxidant system and ROS metabolism, contributing to the antioxidant metabolism insights. The results of the present study fill the knowledge gap on the effect of SA applied by spraying and irrigation on the complex interaction of metabolites and genes in regulating the ripening and senescence processes of pepper fruit. Finally, both foliar spray and irrigation SA applications showed a great effect on fruit quality and crop yield. However, in terms of beneficial effects on the field and for the agri-food industry it should be highlighted the irrigation method due to the easier way and the reduced cost of application compared to the foliar application.

## Data Availability

The original contributions presented in the study are included in the article/**Supplementary Material**, further inquiries can be directed to the corresponding author/s.
