# Supplementary material for: Antioxidant metabolism insights into ripening and senescence delay of green pepper fruit through the salicylic acid preharvest treatment
Source: Front Plant Sci. 2025 Mar 19;16:1475068. doi: 10.3389/fpls.2025.1475068 (PMC11961999; doi:10.3389/fpls.2025.1475068)
Supplement: Supplementary file 1 [file DataSheet1.pdf]

*Supplementary Material*

# Antioxidant Metabolism Insights into Ripening and Senescence Delay of Green Pepper Fruit through the Salicylic Acid Preharvest Treatment

**Alicia Dobón-Suárez[1], María Gutiérrez-Pozo[1], Vicente Serna-Escolano[1], María J. Giménez[1], Daniel Valero[1], María Serrano[2], María E. García-Pastor[2*], Pedro J. Zapata[1]**

[1]Department of Agri-Food Technology, Institute for Agri-Food and Agro-Environmental Research and Innovation (CIAGRO), University Miguel Hernández, Ctra. Beniel km 3.2, 03312, Alicante, Spain

[2]Department of Applied Biology, Institute for Agri-Food and Agro-Environmental Research and Innovation (CIAGRO), University Miguel Hernández, Ctra. Beniel km 3.2, 03312, Alicante, Spain

*** Correspondence:** María E. García-Pastor: m.garciap@umh.es

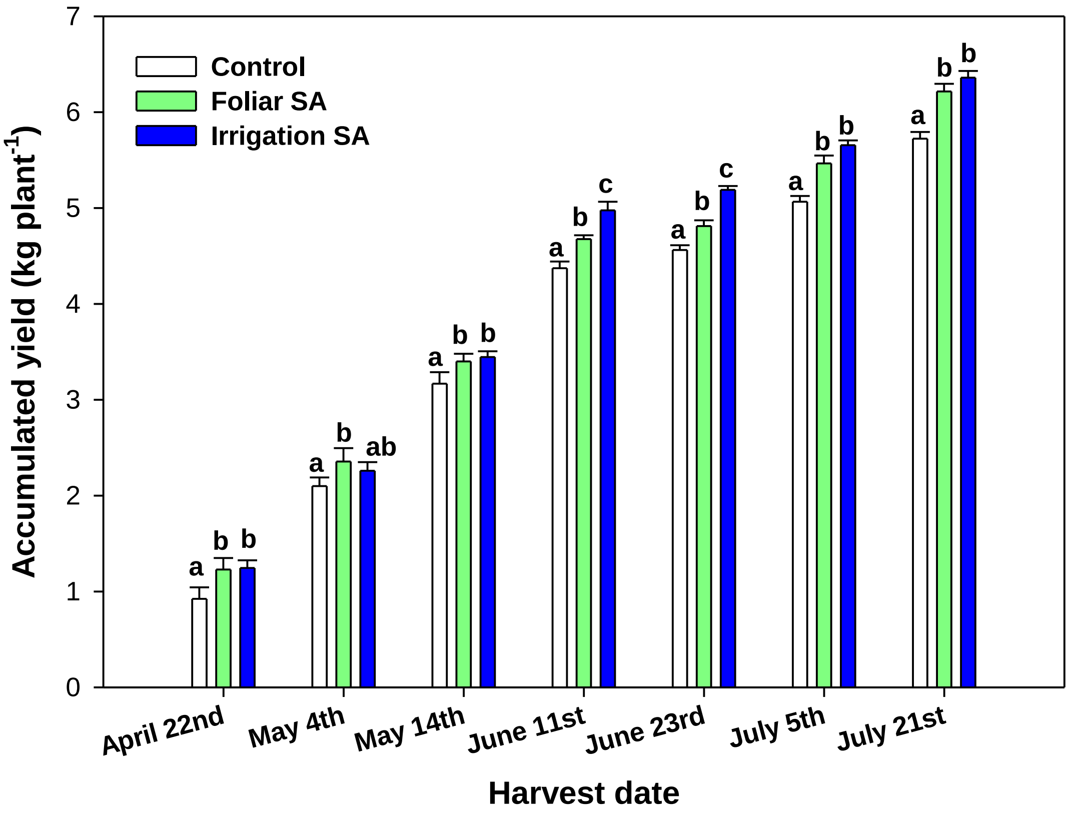


**Supplementary Figure 1.** Accumulated yield (kg plant$^{-1}$) throughout the developmental and growth cycle of green pepper fruit, as affected by salicylic acid (SA) preharvest treatment at 0.5 mM applied by both foliar spraying and irrigation in the 2021 experiment. Data are the mean ± SE. Different lowercase letters show significant differences among treatments for each harvest date at $p < 0.05$.

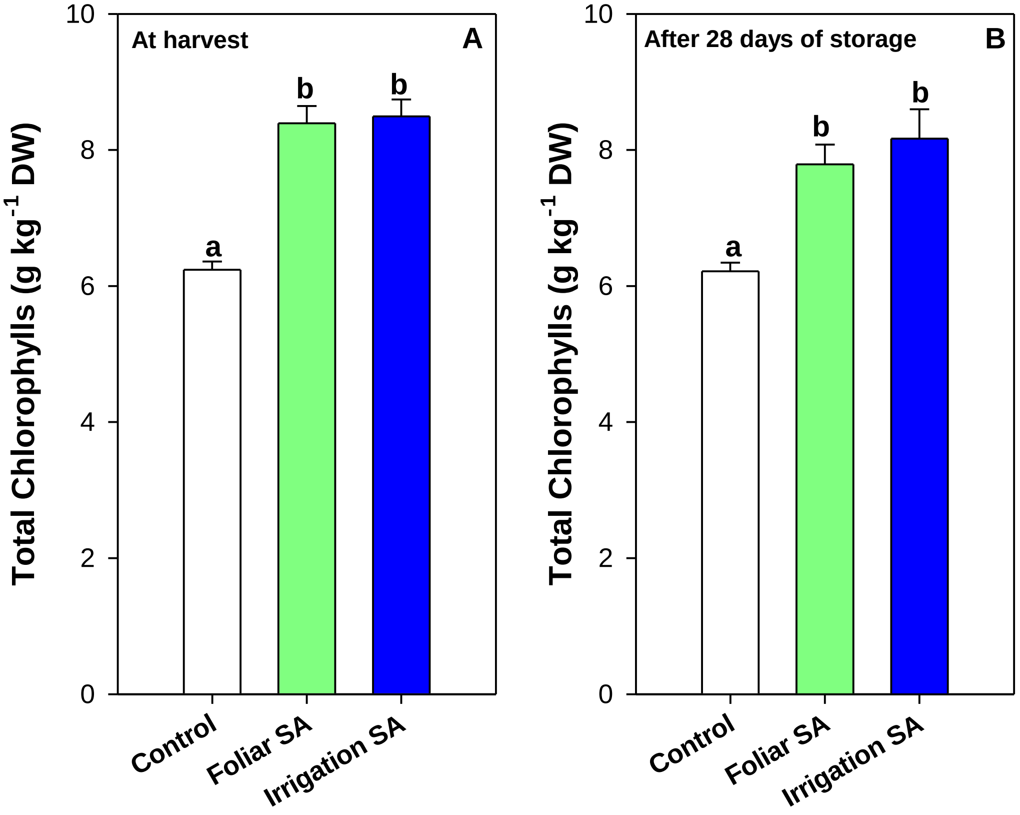


**Supplementary Figure 2.** Effect of salicylic acid (SA) applied by foliar spraying [Foliar SA] and irrigation [Irrigation SA] on total chlorophyll content (g $kg^{-1}$ DW) of green pepper fruit at harvest [**A**] and after 28 days of storage at 7 ºC [**B**]. Data are the mean ± SE. Different lowercase letters show significant differences among treatments for each sampling date at $p < 0.05$.

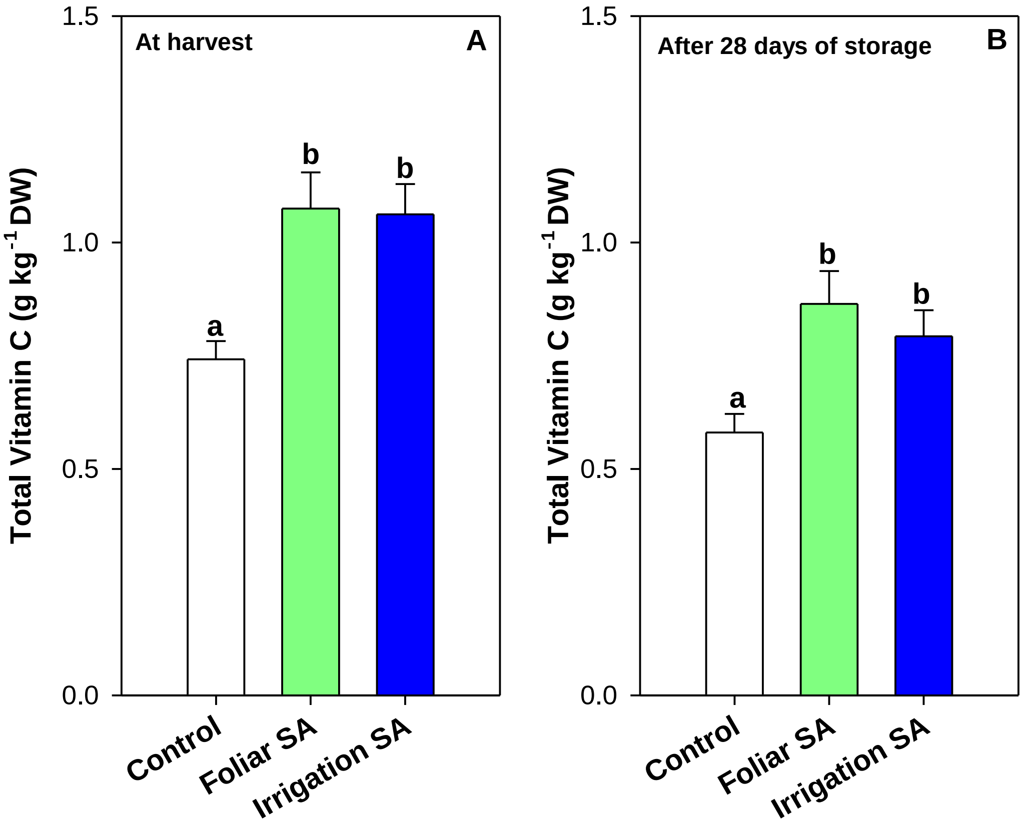


**Supplementary Figure 3.** Effect of salicylic acid (SA) applied by foliar spraying [Foliar SA] and irrigation [Irrigation SA] on total vitamin C content (g $kg^{-1}$ DW) of green pepper fruit at harvest [**A**] and after 28 days of storage at 7 ºC [**B**]. Data are the mean ± SE. Different lowercase letters show significant differences among treatments for each sampling date at $p < 0.05$.

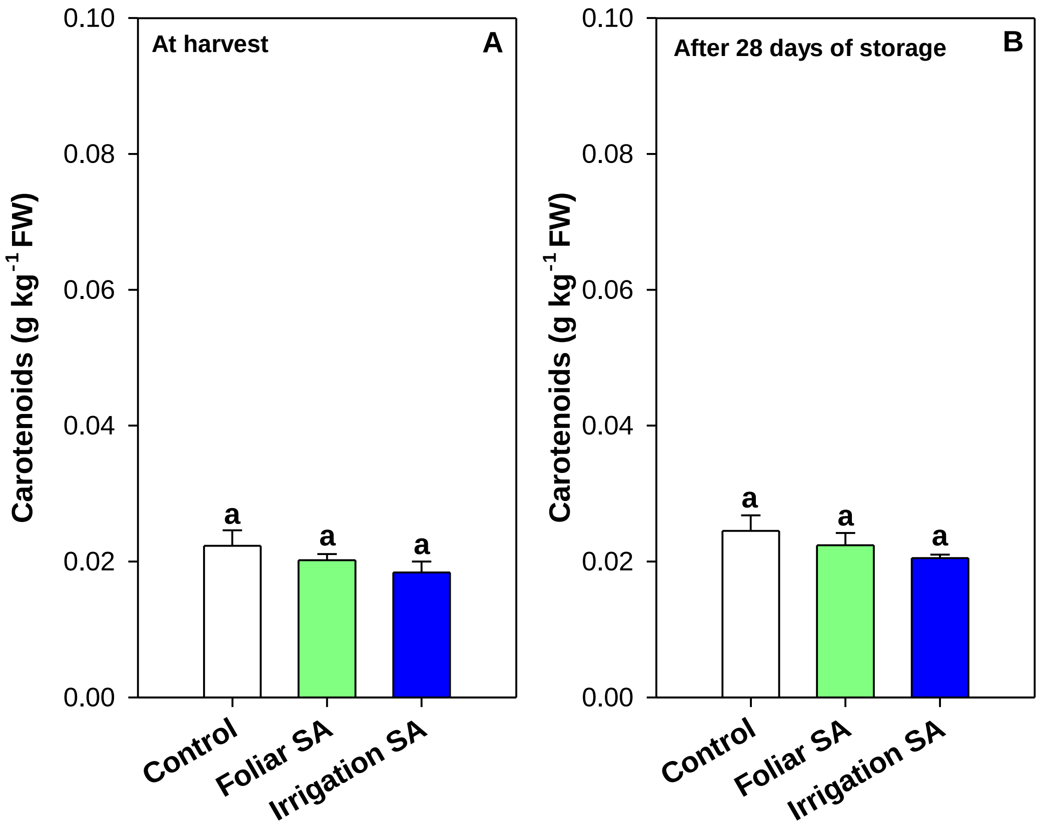


**Supplementary Figure 4.** Effect of salicylic acid (SA) applied by foliar spraying [Foliar SA] and irrigation [Irrigation SA] on carotenoids content (g kg$^{-1}$ FW) of green pepper fruit at harvest [**A**] and after 28 days of storage at 7 ºC [**B**]. Data are the mean ± SE. Different lowercase letters show significant differences among treatments for each sampling date at $p < 0.05$.

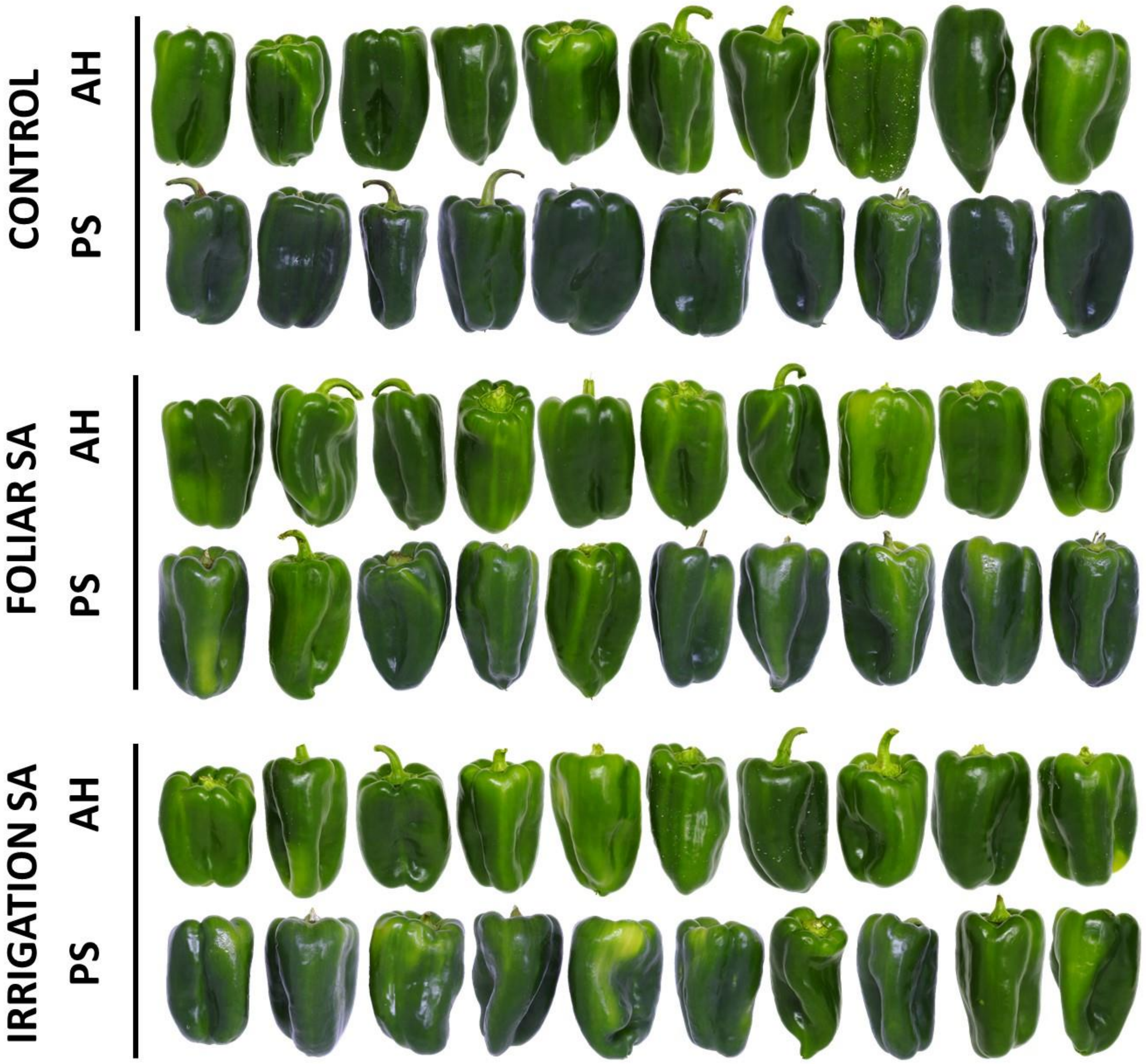


**Supplementary Figure 5.** Effect of control and salicylic acid (SA) applied by foliar spraying [Foliar SA] and irrigation [Irrigation SA] on visual aspect of ‘Lamuyo’ green pepper fruit, ‘Herminio’ cv., at harvest [**AH**] and after 28 days of storage at 7 ºC [**PS**].
